# MnPt@metal organic framework nanozyme with photothermal/catalytic dual-strategy enhances Zn^2+^-mediated immunotherapy through activating pyroptosis and cGAS-STING pathway

**DOI:** 10.1093/rb/rbag210

**Published:** 2026-09-21

**Authors:** Zekai Li, Qi Zhao, Liye Wang, Zhou Shen, Feng Liu, Jiamei Ma, Lining Zhao, Xiaoping Yin, Leyong Zeng

**Affiliations:** College of Chemistry and Materials Science & College of Life Sciences, Chemical Biology Key Laboratory of Hebei Province, Hebei Research Center of the Basic Discipline of Synthetic Chemistry, Hebei University, Baoding 071002, China; Hebei Key Laboratory of Precise Imaging of Inflammation Related Tumors & Department of Medical Oncology & Department of Radiology, Central Laboratory, Affiliated Hospital of Hebei University, Baoding 071000, China; College of Chemistry and Materials Science & College of Life Sciences, Chemical Biology Key Laboratory of Hebei Province, Hebei Research Center of the Basic Discipline of Synthetic Chemistry, Hebei University, Baoding 071002, China; Hebei Key Laboratory of Precise Imaging of Inflammation Related Tumors & Department of Medical Oncology & Department of Radiology, Central Laboratory, Affiliated Hospital of Hebei University, Baoding 071000, China; College of Chemistry and Materials Science & College of Life Sciences, Chemical Biology Key Laboratory of Hebei Province, Hebei Research Center of the Basic Discipline of Synthetic Chemistry, Hebei University, Baoding 071002, China; Hebei Key Laboratory of Precise Imaging of Inflammation Related Tumors & Department of Medical Oncology & Department of Radiology, Central Laboratory, Affiliated Hospital of Hebei University, Baoding 071000, China; College of Chemistry and Materials Science & College of Life Sciences, Chemical Biology Key Laboratory of Hebei Province, Hebei Research Center of the Basic Discipline of Synthetic Chemistry, Hebei University, Baoding 071002, China; Hebei Key Laboratory of Precise Imaging of Inflammation Related Tumors & Department of Medical Oncology & Department of Radiology, Central Laboratory, Affiliated Hospital of Hebei University, Baoding 071000, China; College of Chemistry and Materials Science & College of Life Sciences, Chemical Biology Key Laboratory of Hebei Province, Hebei Research Center of the Basic Discipline of Synthetic Chemistry, Hebei University, Baoding 071002, China; Hebei Key Laboratory of Precise Imaging of Inflammation Related Tumors & Department of Medical Oncology & Department of Radiology, Central Laboratory, Affiliated Hospital of Hebei University, Baoding 071000, China; Key Laboratory of Medicinal Chemistry and Molecular Diagnosis of the Ministry of Education, State Key Laboratory of New Pharmaceutical Preparations and Excipients, Hebei University, Baoding 071002, China

**Keywords:** nanozymes, zinc ion, pyroptosis, cGAS-STING pathway, immunotherapy

## Abstract

Zinc ions (Zn^2+^) can induce immune response through activating pyroptosis and cyclic GMP-AMP synthase (cGAS)-stimulator of interferon genes (STING) pathway, and disrupting the intracellular redox balance is an important strategy for enhancing Zn^2+^-driven immunotherapy. Herein, using bimetallic manganese platinum (MnPt) nanozyme as the core, a multifunctional MnPt@ZIF-8/β-Lap@F-127 nanozyme with multi-enzyme activity was developed by coating metal-organic framework (ZIF-8), carrying β-Lapachone (β-Lap) and modifying F-127 and the Zn^2+^-induced immunotherapy was enhanced through photothermal/catalytic dual-strategy. The MnPt@ZIF-8/β-Lap@F-127 nanozyme has the glutathione peroxidase/peroxidase/catalase-like activity, and the photothermal and photoacoustic imaging properties. Under acidic microenvironment, the MnPt@ZIF-8/β-Lap@F-127 nanozyme was degraded, and the Zn^2+^ and β-Lap were released. By producing a large amount of reactive oxygen species, the MnPt@ZIF-8/β-Lap@F-127 nanozyme induced apoptosis and pyroptosis, and activated the cGAS-STING pathway. Under the dual action of photothermal/catalytic, the mitochondrial function was destroyed and the damage-associated molecular patterns were released, effectively triggering the immunogenic cell death. By improving the immune cells infiltration and pro-inflammatory factors level and reducing the immunosuppressive cells proportion, the MnPt@ZIF-8/β-Lap@F-127 nanozyme greatly inhibited the primary tumor growth and lung metastasis, which demonstrated that the MnPt@ZIF-8/β-Lap@F-127 nanozyme remodeled the immune microenvironment and enhanced the Zn^2+^-induced immunotherapy.

## Graphical Abstract

**Graphical Abstract:**
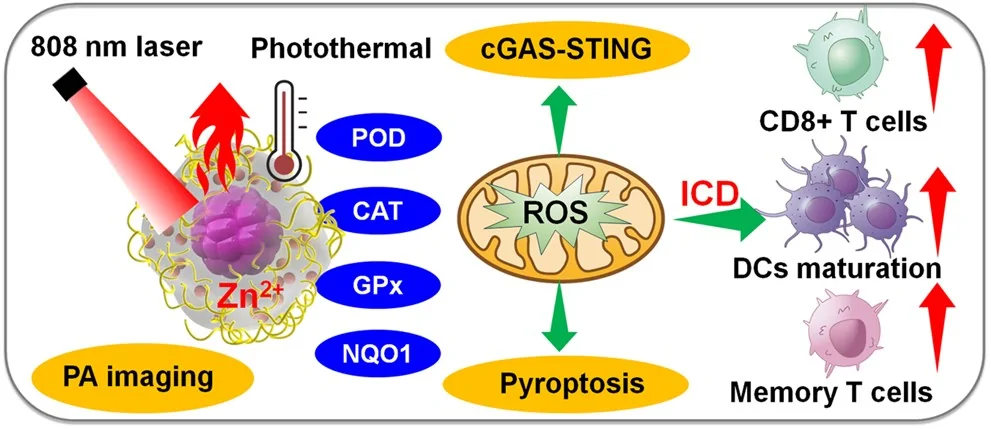

## Introduction

Cancers pose a major social, public health and economic challenge [1], but the traditional treatments lead to significant side effects, also immune escape [2]. Recently, immunotherapy has received widespread attention, which can inhibit tumor by regulating immune system [3, 4]. Owing to the discovery of multiple immune checkpoint inhibitors, immune checkpoint inhibition (ICB) therapy greatly promoted the development of anti-tumor immunotherapy [5, 6]. However, due to immunosuppressive tumor microenvironment (TME) and heterogeneity of tumors, ICB therapy has failed to achieve good efficacy for all types of solid tumors [7]. Remodeling the immune microenvironment is a promising strategy for cancer immunotherapy, thereby activating systemic immunity [8].

Using the excellent enzyme-like activity, nanozyme was widely used as a carrier to reverse immunosuppressive TME [9, 10]. To date, a lot of efforts have been contributed to nanozyme-induced immunogenic cell death (ICD) in tumors, such as cuproptosis, ferroptosis and pyroptosis [11–13]. Among them, pyroptosis is a programmed cell death mediated by gasdermin D (GSDMD), which can activate adaptive immunity [14]. Moreover, by reasonably designing nanozymes, the pyroptosis can precisely be stimulated [15, 16]. In addition, adaptive immunity depends on the participation of innate immunity [17]. As a key mechanism to initiate innate immune defense, cyclic GMP-AMP synthase (cGAS)-stimulator of interferon genes (STING) pathway has been used in tumor immunotherapy [18, 19]. However, the clinical STING agonists have shown disappointing efficacy, owing to their short half-life [20]. Fortunately, owing to the strong reactive oxygen species (ROS) generation and ion interference capability, metallic nanozymes are expected to activate cGAS-STING pathway for simultaneously enhancing innate immunity and adaptive immunity.

Presently, Mn^2+^ and Zn^2+^-mediated nanozymes represent promising therapeutic modalities for activating cGAS-STING pathway [21–23]. A manganese galvanic cell has been engineered to sustainably release Mn^2+^ for triggering immune response activation of the cGAS-STING cascade [24]. Nanoscale metal-organic frameworks (MOFs) are extensively exploited for drug delivery, owing to their porous architectures and stimuli-responsive characteristics [25]. As a typical acid-labile MOF carrier, ZIF-8 has been functionalized to load ferroptosis initiator and single-atom manganese, in which ferroptotic effect and Mn^2+^ output synergistically stimulate STING signaling, and cooperatively remodel the tumor immune microenvironment [26]. Beyond its role as a delivery carrier, Zn^2+^ liberated from acid-triggered ZIF-8 degradation is capable of activating both pyroptosis and the cGAS-STING axis [27, 28]. Analogous to ferroptosis, pyroptosis is classified as a form of ICD, which elicits robust antitumor immune reactions, when coupled with cGAS-STING activation [29]. Therefore, by combining the abundant ROS generation and photothermal performance of metal nanozymes with Zn^2+^ dyshomeostasis of ZIF-8, the combination regimens jointly disrupt cellular homeostasis and have emerged as attractive approaches to synergistically boost antitumor immunotherapy efficacy [30–32].

Herein, using bimetallic manganese/platinum (MnPt) nanozyme as core, a multifunctional MnPt@ZIF-8/β-Lap@F-127 nanozyme was constructed by coating with ZIF-8, loading β-Lapachone (β-Lap) and modifying F-127, by which Zn^2+^-driven antitumor immunotherapy was enhanced through activating pyroptosis and cGAS-STING pathway. As shown in Figure 1, MnPt@ZIF-8/β-Lap@F-127 nanozyme possessed multi-enzyme activities of peroxidase (POD), catalase (CAT) and glutathione peroxidase (GPx), photothermal conversion and photoacoustic (PA) imaging performances, as well as hydrogen peroxide (H_2_O_2_) production capability [33], in which the ROS production was greatly amplified by combining with Zn^2+^-induced mitochondria damage [34]. Moreover, by activating caspase-1 to form GSDMD N-terminal (GSDMD-N), Zn^2+^ can activate the cGAS-STING pathway and induce pyroptosis through photothermal/catalytic dual-strategy. Thus, the developed MnPt@ZIF-8/β-Lap@F-127 nanozyme enhanced Zn^2+^-mediated immunotherapy against primary tumor and lung metastasis of breast cancer.

**Figure 1:**
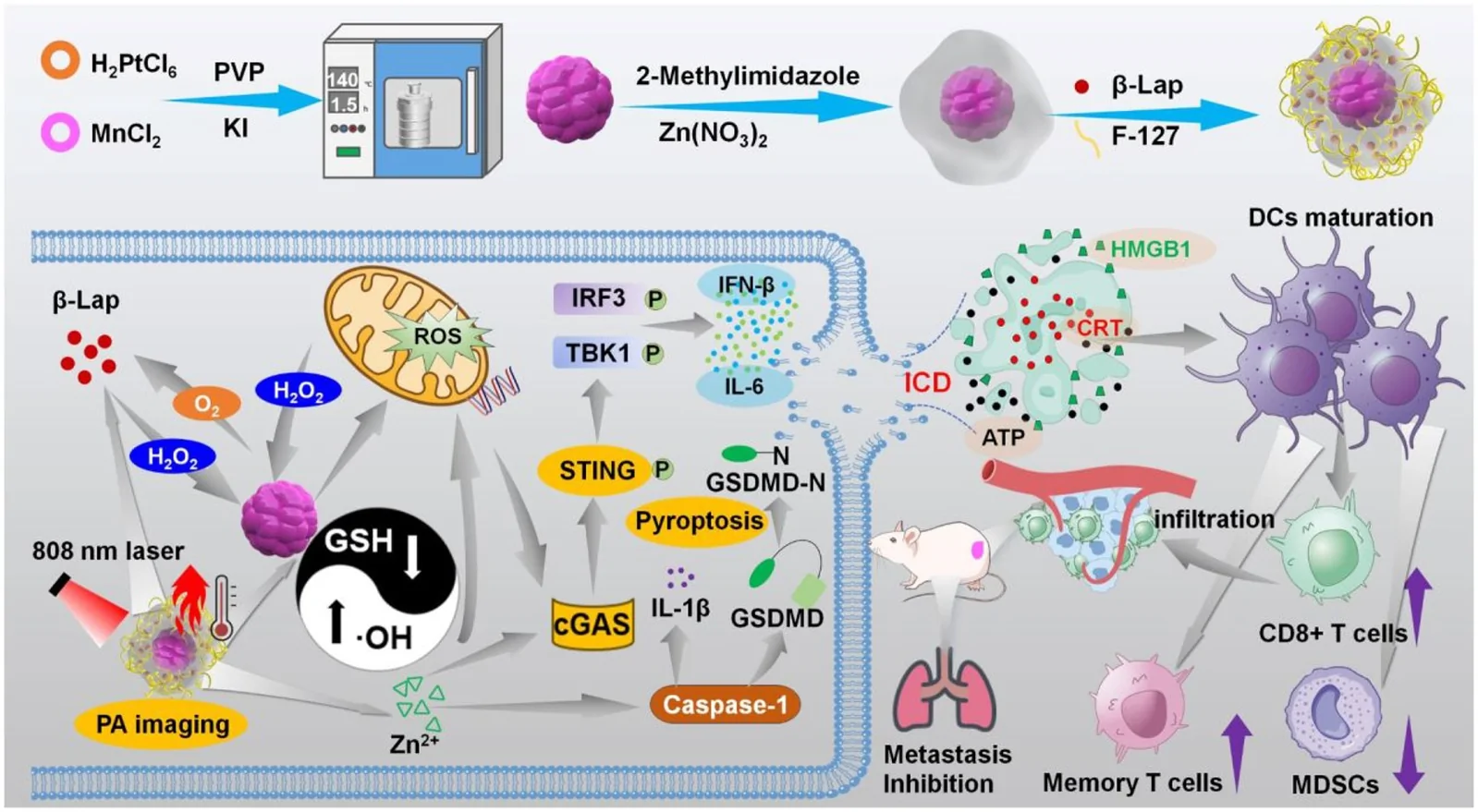
Schematic illustration of MnPt@ZIF-8/β-Lap@F-127 nanozyme with photothermal/catalytic dual-strategy for enhancing Zn^2+^-mediated immunotherapy through activating pyroptosis and cGAS-STING pathway.

## Experimental section

### Materials

Chloroplatinic acid (H_2_PtCl_6_·6H_2_O, ≥99%), potassium iodide (KI, ≥99%), manganese chloride (MnCl_2_, ≥99%), Zincon, β-Lapachone (β-Lap, ≥99%), bovine serum albumin (BSA, ≥95%), 3,3′,5,5′-tetramethylbenzidine (TMB, ≥99%), glutathione (GSH, ≥98%), H_2_O_2_, 2-methylimidazole, 5,5'-dithiobis-(2-nitrobenzoic acid) (DTNB, ≥98%), thiazolyl blue tetrazolium bromide (MTT, 98%), Pluronic F-127 (≥99%), polyvinylpyrrolidone (PVP, MW = 30 000), sodium hydroxide (NaOH, ≥99%) and dimethyl sulfoxide (DMSO, ≥99%) were obtained from Aladdin (Shanghai, China). Calcein AM/PI (CAM/PI), reduced GSH and ATP assay kits were obtained from Beyotime (Beijing, China). Mitochondrial membrane potential (MMP) assay kit (JC-1), Hoechst 33342, Triton X-100, Collagenase IV, DNase I, Annexin V-FITC/PI apoptosis assay kit, ROS assay kit (DCFH-DA), dicoumarol (DIC), protein-free rapid blocking solution and red blood cell lysis buffer were obtained from Solarbio (Beijing, China). Fetal bovine serum (FBS) and culture medium were obtained from Meilunbio (Dalian, China). The antibodies [anti-calreticulin (CRT), anti-high mobility group box 1 (HMGB1), anti-caspase-1 and Cy3 conjugated goat anti-rabbit IgG (H + L)] were obtained from Servicebio (Wuhan, China). The antibodies (anti-p-STING, anti-p-TBK1, anti-p-IRF3 and anti-GSDMD) were obtained from Immunoway (Jiangsu, China). The antibodies (anti-CD80, anti-CD86, anti-CD11c, anti-CD11b, anti-Ly6G, anti-Ly6C, anti-CD45, anti-CD8, anti-CD62L, anti-CD44, anti-CD3 and anti-CD4) were obtained from Elabscience (Wuhan, China).

### Synthesis of MnPt@ZIF-8/β-Lap@F-127

#### Synthesis of MnPt

Five milligrams of MnCl_2_ and 20.8 mg of H_2_PtCl_6_·6H_2_O were dissolved in 10 mL of H_2_O. After stirring for 10 min, 50 μL of KI (0.8 g/mL) and 1.6 mL of PVP (0.2 g/mL) were added with thorough stirring. Then, they were transferred into a reactor kettle, and the reaction was maintained at 140°C for 90 min. After cooling to room temperature, 10 mL of acetone was added, and the mixture was allowed to stand for 8 h. Finally, the obtained MnPt was centrifuged and washed.

#### Synthesis of MnPt@ZIF-8

In a methanol system, 1 mL of Zn(NO_3_)_2_·6H_2_O (11 mg/mL) and 1 mL of 2-methylimidazole (22 mg/mL) were mixed with 10 mL of MnPt (1 mg/mL). After stirring for 40 min, the MnPt@ZIF-8 was collected through centrifugation and washing.

#### Synthesis of MnPt@ZIF-8/β-Lap@F-127

In a methanol system, 400 μL of β-Lap (10 mg/mL) was slowly added to 10 mL of MnPt@ZIF-8 (0.1 mg/mL) and stirred for 30 min. After adding 1 mL of F-127 (5 mg/mL), they were stirred for 30 min, and the MnPt@ZIF-8/β-Lap@F-127 was centrifuged and washed.

### Characterization

Morphology was measured on a Tecnai F30 transmission electron microscope (FEI, USA). Ultraviolet–Visible (UV–Vis) spectra were obtained on a TU-1950 UV–Vis spectrophotometer (Persee, China). Statistical size and potential were characterized on a ZEN3700 zeta-sizer (Malvern, UK). X-ray diffraction (XRD) spectra were collected on a D8 Advance X-ray diffractometer (Bruker, Germany). Chemical composition was analyzed with an Axis Ultra DLD X-ray photoelectron spectrometer (Kratos, UK).

### pH-sensitive release of β-Lap and Zn^2+^

#### Release of β-Lap

Three milliliters of MnPt@ZIF-8/β-Lap@F-127 (1 mg/mL) in dialysis bag were immersed in 20 mL of PBS (pH = 5.5, 6.5 and 7.4), respectively. At 1, 2, 4, 8, 16, 26 and 36 h, 1 mL of PBS was extracted and 1 mL of fresh PBS was added. Finally, the β-Lap absorbance of extracted PBS was recorded using a UV–Vis spectrometer.

#### Release of Zn^2+^

Five  milliliters of MnPt@ZIF-8/β-Lap@F-127 (1 mg/mL) in dialysis bag were immersed in 20 mL of PBS (pH = 5.5, 6.5 and 7.4). At 0.5, 1, 2, 4, 8, 12 and 24 h, 1 mL of PBS was extracted and 1 mL of fresh PBS was added. Then, 50 µL of the extracted PBS containing Zn^2+^ was mixed with 100 µL of zincon. Finally, the absorbance was recorded on a microplate reader.

### Photothermal, GSH consumption and ·OH/O_2_ generation measurement

#### Photothermal measurement

One milliliter of MnPt@ZIF-8/β-Lap@F-127 (0, 50, 100, 150 and 200 μg/mL) was subjected to 808 nm laser irradiation (1.2 W/cm^2^, 10 min), and an infrared thermal imager was used to record the temperature change. Then, the influence of power density (0.2, 0.4, 0.6, 0.8, 1.0 and 1.2 W/cm^2^) on the temperature change was investigated. Furthermore, the photothermal stability was assessed through five cycles of heating and cooling, and the photothermal conversion efficiency was calculated based on the heating and cooling processes.

#### GSH consumption

DTNB was utilized to measure GSH consumption, in which GSH reacted with DTNB to form yellow thionitrobenzoic acid (TNB). In a PBS system (pH = 6.5), 940 μL of MnPt@ZIF-8/β-Lap@F-127 (0, 50, 100, 150, 200 and 250 μg/mL) and 60 μL of GSH (100 mM) were incubated at 37°C for 8 h. Then, the supernatant was collected through centrifugation, and the remaining GSH content in the supernatant was assessed by measuring the TNB absorbance. Additionally, the effect of incubation time (2, 4, 6 and 8 h) and temperature (25, 37 and 49°C) on the GSH consumption of MnPt@ZIF-8/β-Lap@F-127 (200 μg/mL) was characterized.

#### ·OH generation

TMB was utilized to detect the ·OH generation. In a PBS system (pH = 6.5), 1 mL of MnPt@ZIF-8/β-Lap@F-127 (0, 50, 100, 150, 200 and 250 μg/mL) containing GSH (7 mM) and H_2_O_2_ (10 mM) was mixed with 10 μL of TMB (10 mg/mL). After incubation at 37°C for 30 min, the mixture was centrifuged, and the absorbance of the supernatant in each group was measured. Additionally, the effect of GSH concentrations (1, 2, 3, 4, 5, 6, 7 and 8 mM) and H_2_O_2_ (6, 8, 10 and 12 mM) on the ·OH formation of MnPt@ZIF-8/β-Lap@F-127 (200 μg/mL) was assessed.

#### O_2_ generation

A dissolved oxygen meter was utilized to assess the O_2_ production capacity. In a PBS system (pH = 6.5), 40 μL of H_2_O_2_ (100 mM) was added into 2 mL of MnPt@ZIF-8/β-Lap@F-127 (0, 50, 100 and 200 μg/mL), and the change of O_2_ content was recorded and compared. Similarly, the effect of H_2_O_2_ concentrations (0.5, 1, 2 and 4 mM) on the O_2_ production of MnPt@ZIF-8/β-Lap@F-127 (200 μg/mL) was also evaluated.

### Cytotoxicity, cellular uptake and intracellular ROS/O_2_/GSH detection

#### Cytotoxicity

Cytotoxicity was measured using MTT assay. 1 × 10^4^ MCF-10A cells were cultured for 24 h. Subsequently, 100 μL of culture medium containing MnPt, MnPt@ZIF-8 and MnPt@ZIF-8/β-Lap@F-127 (0, 50, 100, 150 and 200 μg/mL) was added, and the cells were co-incubated for an additional 20 h. After this incubation period, the culture medium was removed, and 10 μL of MTT (5 mg/mL) was introduced to the wells for a 4 h incubation. Following this, the supernatant was discarded, and 150 μL of DMSO was added to each well. After a 15-min shaking period, the absorbance was recorded on a Synergy HTX microplate reader (BioTek, USA).

#### Cellular uptake

MnPt@ZIF-8/β-Lap@F-127 was labeled with rhodamine B (RhB) for subsequent experiments. 4T1 cells (1 × 10^4^) were cultured for 24 h. Then, the RhB-modified MnPt@ZIF-8/β-Lap@F-127 (200 μg/mL) was incubated with the cells for 0, 2 and 4 h. Finally, the cellular uptake was assessed by measuring the RhB fluorescence intensity using an LSM-880 confocal microscope (Zeiss, Germany).

#### Intracellular ROS

The DCFH-DA fluorescent probe was employed to characterize the ROS production capacity in cells. 4T1 cells (1 × 10^4^) were incubated with 100 μL of MnPt, MnPt@ZIF-8, MnPt@ZIF-8/β-Lap@F-127 and MnPt@ZIF-8/β-Lap@F-127 + DIC (200 μg/mL) for 4 h. Alternatively, the cells in the MnPt@ZIF-8/β-Lap@F-127 and MnPt@ZIF-8/β-Lap@F-127 + DIC were irradiated (1.0 W/cm^2^) for 5 min. Subsequently, 100 μL of the DCFH-DA working solution was added into the cells in the five groups to incubate for 30 min. Finally, the cells were stained with Hoechst 33342 for 30 min, and the fluorescence images were captured using a confocal microscope. Moreover, 1 × 10^5^ 4T1 cells were treated by following the above procedures, by which the statistical ROS generation was measured on a CytoFLEX S flow cytometer (Beckman Coulter, USA).

#### Intracellular O_2_

The intracellular O_2_ generation and consumption were quantified using the RDPP fluorescent probe. 4T1 cells (1 × 10^4^) were incubated with 100 μL of MnPt, MnPt@ZIF-8 and MnPt@ZIF-8/β-Lap@F-127 (200 μg/mL) for 4 h. Alternatively, the cells in the MnPt@ZIF-8/β-Lap@F-127 were irradiated (1.0 W/cm^2^) for 5 min. Subsequently, the cells in the five groups were incubated with RDPP for 20 min and stained with Hoechst for 30 min. Finally, the red fluorescence was observed using a confocal microscope.

#### Intracellular GSH

A GSH content assay kit was utilized to measure the intracellular GSH concentration. 4T1 cells (1 × 10^5^) were incubated with 100 μL of MnPt@ZIF-8/β-Lap@F-127 (0, 50, 100, 150 and 200 μg/mL) for 4 h. Then, the cells were collected, lysed and 180 μL of the supernatant was incubated with 20 μL of GSH detection working solution. Finally, the absorbance at 412 nm was measured to calculate the GSH consumption.

### Intracellular ATP/Zn^2+^ and mitochondrial function detection

#### Intracellular ATP

An ATP assay kit was employed to measure the intracellular ATP release. 4T1 cells (1 × 10^5^) were incubated with 100 μL of MnPt, MnPt@ZIF-8 and MnPt@ZIF-8/β-Lap@F-127 (200 μg/mL) for 4 h. Alternatively, the cells in the MnPt@ZIF-8/β-Lap@F-127 were irradiated (1.0 W/cm^2^) for 5 min. After incubation for 8 h, the cells in the five groups were centrifuged, and 20 μL of the supernatant was added into 100 μL of ATP detection working solution. Finally, the relative luminescence unit (RLU) value was measured on a microplate reader.

#### Intracellular Zn^2+^

TSQ was utilized as a fluorescent probe to monitor the intracellular Zn^2+^ concentration. 4T1 cells (1 × 10^4^) were incubated with 100 μL of MnPt, MnPt@ZIF-8 and MnPt@ZIF-8/β-Lap@F-127 (200 μg/mL) for 4 h. Then, 100 μL of TSQ (30 μM) was added for an additional 30 min incubation. Finally, they were stained with FITC-labeled phalloidin, and the fluorescence intensity of TSQ was observed on a confocal microscope.

#### Mitochondrial function

The JC-1 assay kit was employed to assess the MMP reduction induced by mitochondrial damage. 4T1 cells (1 × 10^4^) were incubated with 100 μL of MnPt, MnPt@ZIF-8 and MnPt@ZIF-8/β-Lap@F-127 (200 μg/mL) for 4 h. Alternatively, the cells in the MnPt@ZIF-8/β-Lap@F-127 were irradiated (1.0 W/cm^2^) for 5 min. Subsequently, the cells in the five groups were incubated with the JC-1 staining solution at 37°C for 20 min. Finally, they were washed with JC-1 staining buffer, and the red/green fluorescence was observed using a confocal microscope. In addition, the JC-1 fluorescence was also statistically analyzed using flow cytometry, in which 1 mL of 4T1 cells (1 × 10^5^ cells/mL) were treated with different samples by following the above procedures, and the cells were detected using a flow cytometer.

### Pyroptosis and cGAS-STING pathway detection

First, 1 × 10^4^ 4T1 cells were incubated for 24 h. Then, the culture medium was replaced with 200 μL of MnPt, MnPt@ZIF-8, MnPt, MnPt@ZIF-8, and MnPt@ZIF-8/β-Lap@F-127 (200 μg/mL) for 4 h incubation. Alternatively, the cells in the MnPt@ZIF-8/β-Lap@F-127 were irradiated (1.0 W/cm^2^) for 5 min. After fixation with paraformaldehyde, 100 μL of the antibodies (including p-STING, p-TBK1, p-IRF3, GSDMD-N, CRT and HMGB1) was, respectively, added and incubated at 4°C for 8 h in the dark. Then, 100 μL of Cy3-labeled goat anti-rabbit IgG was added and incubated at 37°C for 2 h in the dark. Finally, the cells were stained with Hoechst and observed on a confocal microscope.

### Cell apoptosis detection

#### MTT assay

4T1 cells (1 × 10^4^) were cultured for 24 h. Subsequently, 100 μL of MnPt, MnPt@ZIF-8 and MnPt@ZIF-8/β-Lap@F-127 (0, 50, 100, 150 and 200 μg/mL) was added and co-cultured for 4 h. Alternatively, the cells in the MnPt@ZIF-8/β-Lap@F-127 were irradiated (1.0 W/cm^2^) for 5 min. After an additional 16 h incubation, 10 μL of MTT (5 mg/mL) was added for 4 h incubation, and the absorbance was recorded on a microplate reader.

#### CAM/PI staining

First, 1 × 10^4^ 4T1 cells were cultured for 24 h. Then, the culture medium was replaced with 200 μL of MnPt, MnPt@ZIF-8, MnPt, MnPt@ZIF-8, and MnPt@ZIF-8/β-Lap@F-127 (200 μg/mL). Alternatively, the cells in the MnPt@ZIF-8/β-Lap@F-127 were irradiated (1.0 W/cm^2^) for 5 min. After another 8 h incubation, 200 μL of CAM/PI working solution was added and incubated at 37°C for 30 min. Finally, the red/green fluorescence was observed under an Axio Vert.A1 inverted fluorescence microscope (Zeiss, Germany).

#### Flow cytometry

4T1 cells (1 × 10^5^) were cultured for 24 h. Then, the culture medium was replaced with 1 mL of MnPt, MnPt@ZIF-8, MnPt@ZIF-8/β-Lap@F-127 and MnPt@ZIF-8/β-Lap@F-127 (200 μg/mL). Alternatively, the cells in the MnPt@ZIF-8/β-Lap@F-127 were irradiated (1.0 W/cm^2^) for 5 min. After another 8 h incubation, the cells were collected and mixed with 5 μL of Annexin V-FITC and 10 μL of PI and incubated for 15 min. Finally, the cell apoptosis was detected on a flow cytometer.

### *In vivo* antitumor immunotherapy

BALB/c female mice were used in the experiments, which was purchased from Vital River (Beijing, China). All the experimental protocols were approved by the Animal Welfare and Ethical Committee of Hebei University (Approval No. IACUC-HBU2025M074), and all animal measurements and pathological analyses were performed in a blinded manner. To establish the 4T1 primary tumor and lung-metastatic tumor models, 1 × 10^6^ 4T1 cells were subcutaneously injected into BALB/c mice. After 10 days, 5 × 10^5^ 4T1 cells were injected into BALB/c mice via tail vein.

#### Biocompatibility

To investigate the blood biocompatibility, the hemolysis rate of MnPt@ZIF-8/β-Lap@F-127 (50, 100, 150, 200, 300, 400 and 500 μg/mL) was measured by following a previously reported method [35]. To verify the biological safety, healthy mice were injected with 100 μL of saline and MnPt@ZIF-8/β-Lap@F-127 (500 μg/mL) via tail vein, respectively. After 14 days, the mice were sacrificed, the blood was taken to test blood routine and biochemistry, and the heart, liver, spleen, lung and kidney were taken to make H&E staining for observing organ lesions.

#### *In vivo* PA imaging

When the primary tumor grew to 50–100 mm^3^, the PA imaging was performed. One hundred microliters of MnPt@ZIF-8/β-Lap@F-127 (500 μg/mL) was injected through tail vein, and the PA images were captured at 0, 1, 2, 4, 8, 12, 24 and 30 h, using a Marsonics-PIIP-FC PA integrated imaging platform (Tsingpai, Beijing) under the excitation of 808 nm laser.

#### *In vivo* combination therapy

The mice were categorized into five groups: Saline, MnPt, MnPt@ZIF-8, MnPt@ZIF-8/β-Lap@F-127 and MnPt@ZIF-8/β-Lap@F-127 with laser. For each group, 100 μL of different samples (500 μg/mL) was injected via tail vein. After 12 h, the tumor in the MnPt@ZIF-8/β-Lap@F-127 with laser was exposed to an 808 nm laser (0.8 W/cm^2^) for 5 min. On Days 1, 4, 7 and 10, the mice were treated for four times. Moreover, the body weight and tumor volume were recorded every other day. After 15 days, the mice were sacrificed for different analyses.

#### Blood and tissue analysis

After treatment, the blood samples in the five groups of G5 group were taken to test the content of immune-related cytokines (including IL-6, IFN-β and IL-1β) using enzyme-linked immunosorbent assay (ELISA) method. For the group of MnPt@ZIF-8/β-Lap@F-127 with laser, the blood biochemistry was measured and the H&E staining of organs (heart, liver, spleen and kidney) was observed.

For the five groups, the tumor sections were treated with H&E, Ki-67 and TUNEL staining for evaluating the tumor inhibition and apoptosis, and the lungs were stained with H&E for observing the tumor lung metastasis. Moreover, the tumors were treated with CRT, HMGB1, Caspase-1, GSDMD-N, p-STING, p-TBK1 and p-IRF3 for evaluating the immune activation pathways.

### Statistical analysis

The data were presented as mean ± standard deviation (SD), and the sample size was not <3. The data were analyzed using GraphPad Prism 10.1.2 software, and an unpaired two-tailed t-test was used to determine the statistical differences, in which the significance level was expressed by the following symbols: * *P* < 0.05, ** *P* < 0.01, *** *P* < 0.001 and **** *P* < 0.0001.

## Results and discussion

### Morphology and basic property characterization

First, the morphology of MnPt, MnPt@ZIF-8 and MnPt@ZIF-8/β-Lap@F-127 was characterized. In the transmission electron microscopy (TEM) images, the MnPt nanoparticles exhibited a flower-like structure with a diameter of ∼20 nm (Figure 2A), and the high-resolution TEM (HRTEM) image confirmed that the lattice spacing of 0.232 nm corresponded to the (111) plane of Pt (Figure 2B), and the lattice spacing of 0.195 nm was slightly smaller than that of the (200) plane of Pt (0.20 nm), which can be attributed to the incorporation of smaller Mn atoms [36]. By coating with ZIF-8 the MnPt@ZIF-8 with core-shell structure was formed (Figure 2C and D). Moreover, by loading β-Lap and modifying F-127, the obtained MnPt@ZIF-8/β-Lap@F-127 showed a similar morphology with MnPt@ZIF-8 (Figure 2E and F), demonstrating the good dispersibility. Furthermore, the elemental mapping images exhibited that the Mn and Pt elements were uniformly distributed in the inner layer (Figure 2G), and the Zn, C, N and O elements were observable throughout the overall MnPt@ZIF-8/β-Lap@F-127 nanoparticle (Figure 2H).

**Figure 2:**
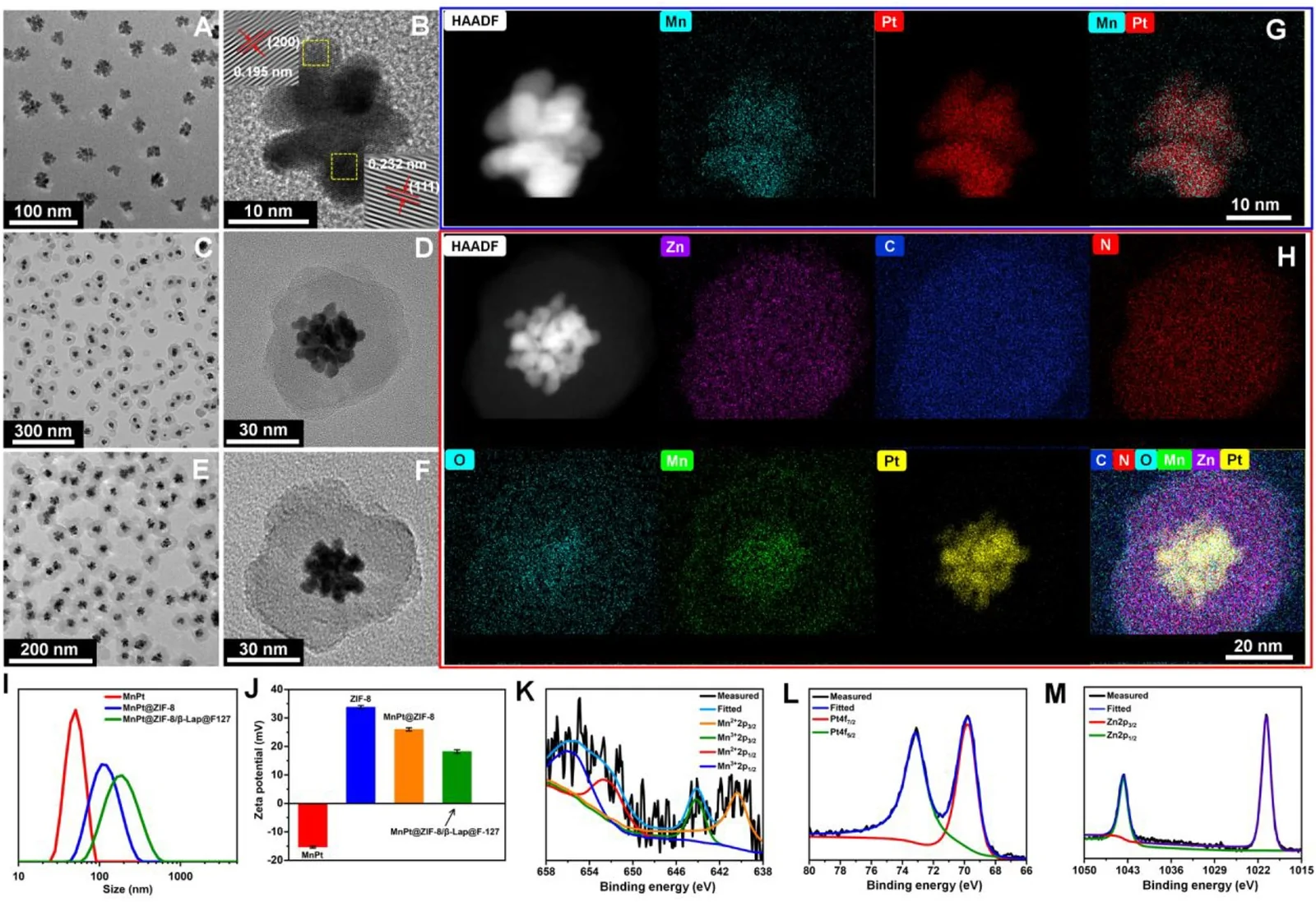
**(A and B)** TEM images of MnPt. (**C and D**) TEM images of MnPt@ZIF-8. (**E and F**) TEM images of MnPt@ZIF-8/β-Lap@F-127. (**G**) TEM mapping images of MnPt. (**H**) TEM mapping images of MnPt@ZIF-8. (**I**) DLS curves. (**J**) Zeta potentials (*n* = 3). (**K**–**M**) XPS spectra of MnPt@ZIF-8/β-Lap@F-127 in the orbits of Mn 2p, Pt 4f and Zn 2p.

Subsequently, the hydrated size and zeta potential were measured. technique, as conducted by a particle size analyzer. As illustrated in the dynamic light scattering (DLS) curves, the average size of MnPt, MnPt@ZIF-8 and MnPt@ZIF-8/β-Lap@F-127 was ∼50, 106 and 190 nm (Figure 2I), and the potential was −15.37, **+**26.83 and **+**18.23 mV (Figure 2J), in which the positive potential was attributed to ZIF-8 and F-127 [37, 38]. By dispersing in different media, the hydration diameter of MnPt@ZIF-8/β-Lap@F-127 did not change significantly over 7 days, demonstrating its colloid stability (Supplementary Figure S1). The elemental composition of MnPt@ZIF-8/β-Lap@F-127 was analyzed using X-ray photoelectron spectroscopy (XPS). The Mn 2p peaks consisted of Mn^2+^ and Mn^3+^, in which the peaks at 652.8 and 640.4 eV belonged to Mn^2+^, and the peaks at 655.7 and 644.2 eV belonged to Mn^3+^ (Figure 2K) [39]. The Pt 4f_5/2_ and Pt 4f_7/2_ peaks were observed at 73.1 and 69.8 eV (Figure 2L), which represented the Pt^0^ [40]. The Zn 2p orbit included the Zn 2p_1/2_ and Zn 2p_3/2_ peaks located at 1043.6 and 1020.6 eV (Figure 2M).

The crystal structure was measured and analyzed (Supplementary Figure S2). The XRD pattern of MnPt revealed five distinct peaks at 39.9°, 46.4°, 67.7° and 81.7°, corresponding to the (111), (200), (220) and (311) planes of MnPt [41]. In the XRD pattern of MnPt@ZIF-8/β-Lap@F-127, some small-angle diffraction peaks were observed at 7.3°, 10.3°, 12.7° and 18.0°, representing the (011), (002), (112) and (222) planes of ZIF-8 [42]. Moreover, a broad absorption band was observed in visible and near infrared (NIR) regions, and the characteristic absorption peak of β-Lap appeared at 430 nm (Figure 3A), demonstrating the NIR absorption capability of MnPt and the successful incorporation of β-Lap. Additionally, in the acidic microenvironment, the ZIF-8 shell gradually collapsed (Supplementary Figure S3), confirming the degradation capability of ZIF-8.

**Figure 3:**
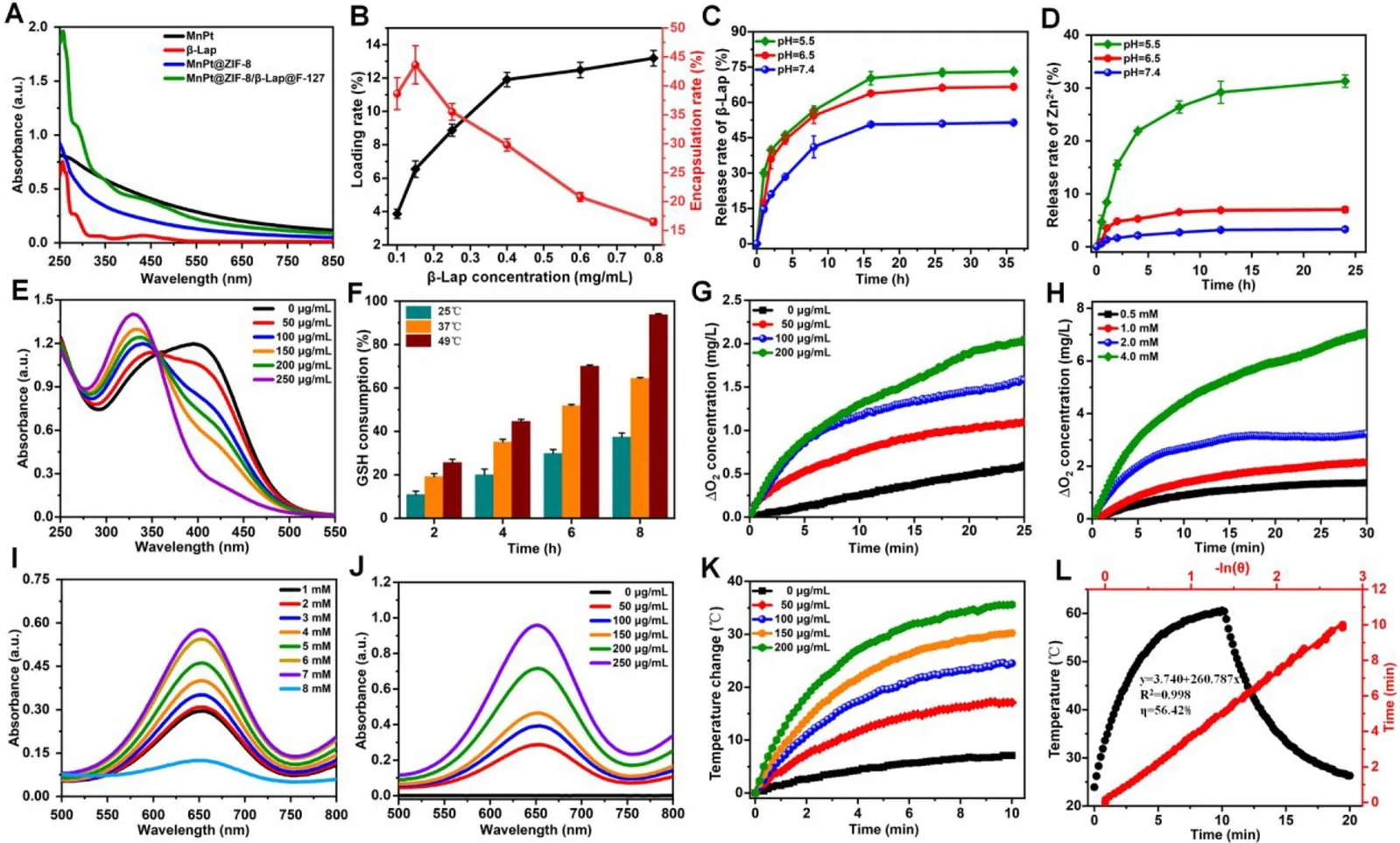
(**A**) UV–Vis absorption spectra. (**B**) Loading/encapsulation curves of β-Lap (*n* = 3). (**C**) β-Lap release curves (*n* = 3). (**D**) Zn^2+^ release curves (*n* = 3). (**E**) Absorbance curves of DTNB incubated with MnPt@ZIF-8/β-Lap@F-127. (**F**) GSH consumption of MnPt@ZIF-8/β-Lap@F-127 (*n* = 3). (**G and H**) O_2_ concentration curves of MnPt@ZIF-8/β-Lap@F-127 with different sample concentrations and different H_2_O_2_ concentrations. (**I and J**) Absorbance curves of TMB incubated with MnPt@ZIF-8/β-Lap@F-127 at different GSH concentrations and different sample concentrations. (**K**) Temperature curves of MnPt@ZIF-8/β-Lap@F-127. (**L**) Photothermal conversion curve of MnPt@ZIF-8/β-Lap@F-127.

### Enzyme-like activity and photothermal performance

By changing the β-Lap concentration, the optimal loading and encapsulation rates were 11.9% and 29.8% (Figure 3B and Supplementary Figure S4). The release properties of β-Lap and Zn^2+^ were investigated. The β-Lap release was greatly promoted by the acidic microenvironment. As depicted in Figure 3C, by reducing the pH from 7.4 and 6.5 to 5.5, the β-Lap release rate within 36 h increased from 51.5% and 66.6% to 73.1%. The ZIF-8 degradation resulted in the Zn^2+^ release, and the pH-responsive Zn^2+^ release was monitored. Similar to the β-Lap release, the Zn^2+^ release was also positively correlated with system acidity. By reducing the pH from 7.4 and 6.5 to 5.5, the Zn^2+^ release rate within 24 h reached 3.3%, 7.0% and 31.3%, respectively (Figure 3D and Supplementary Figure S5), demonstrating the TME-responsive degradation and release capability of MnPt@ZIF-8/β-Lap@F-127.

Due to the presence of Mn^3+^, the GSH consumption capacity of MnPt@ZIF-8/β-Lap@F-127 was assessed, which was determined by observing the reduction of the TNB absorbance at 412 nm. By increasing the MnPt@ZIF-8/β-Lap@F-127 concentration, the TNB absorbance gradually decreased (Figure 3E) and the incubation temperature significantly affected the GSH consumption, in which the GSH consumption rate within 6 h was 37, 64% and 94%, corresponding to the incubation temperature of 24°C, 36°C and 49°C (Figure 3F). MnPt@ZIF-8/β-Lap@F-127 could catalyze H_2_O_2_ to produce O_2_ via CAT-like activity. In the presence of H_2_O_2_, the O_2_ production capacity increased with increasing the MnPt concentration (Figure 3G) and the H_2_O_2_ concentration (Figure 3H). By fixing the H_2_O_2_ concentration (2 mM), the produced O_2_ concentration of MnPt (200 μg/mL) reached 2.3 mg/L, suggesting the excellent CAT-like activity of MnPt@ZIF-8/β-Lap@F-127.

GSH promoted the conversion of Mn^3+^ to Mn^2+^, and the ·OH was formed through Mn^2+^-mediated Fenton-like reaction. The ·OH formation was detected using the TMB probe, which was determined by observing the increased absorbance. By increasing the GSH level, the TMB absorbance initially increased but decreased with GSH concentration (>7 mM; Figure 3I), because the extra GSH could consume the produced ·OH. Moreover, by increasing the sample and H_2_O_2_ concentrations, the TMB absorbance rose obviously (Figure 3J and Supplementary Figure S6), confirming the ·OH production capacity of MnPt@ZIF-8/β-Lap@F-127.

Under an 808 nm laser irradiation, the photothermal property was measured. Within 10 min, the temperature of MnPt@ZIF-8/β-Lap@F-127 (200 μg/mL) increased by 35.6°C (Figure 3K), and the laser power greatly affected the temperature change (Supplementary Figure S7). Through five cycles of on/off laser, the temperature change remained consistent (Supplementary Figure S8), demonstrating the remarkable photothermal stability. By following the heating and cooling process (Figure 3L), the photothermal conversion efficiency of MnPt@ZIF-8/β-Lap@F-127 was calculated to be 56.42%.

### Intracellular enzyme-like activity and *in vitro* efficacy

Zn^2+^ can induce the immune activation via pyroptosis and cGAS-STING pathway, then the ROS production and photothermal effect of MnPt@ZIF-8/β-Lap@F-127 nanozyme can further enhance this process (Figure 4A). Mitochondrial damage caused mitochondrial DNA release, which together with Zn^2+^ constituted a recognition factor for cGAS and synthesize cGAMP. The combination of cGAMP and STING on the endoplasmic reticulum ultimately promoted the IRF3 phosphorylation through a cascade reaction. Phosphorylated IRF3 translocated into the nucleus, initiated type I interferon expression and activated downstream immune responses. Moreover, owing to thermal effect and Zn^2+^-induced pyroptosis, GSDMD was cleaved by caspase-1 to generate GSDMD-N, which eventually led to the formation of cell membrane pores and the release of cell contents to the extracellular space to induce immune activation.

**Figure 4:**
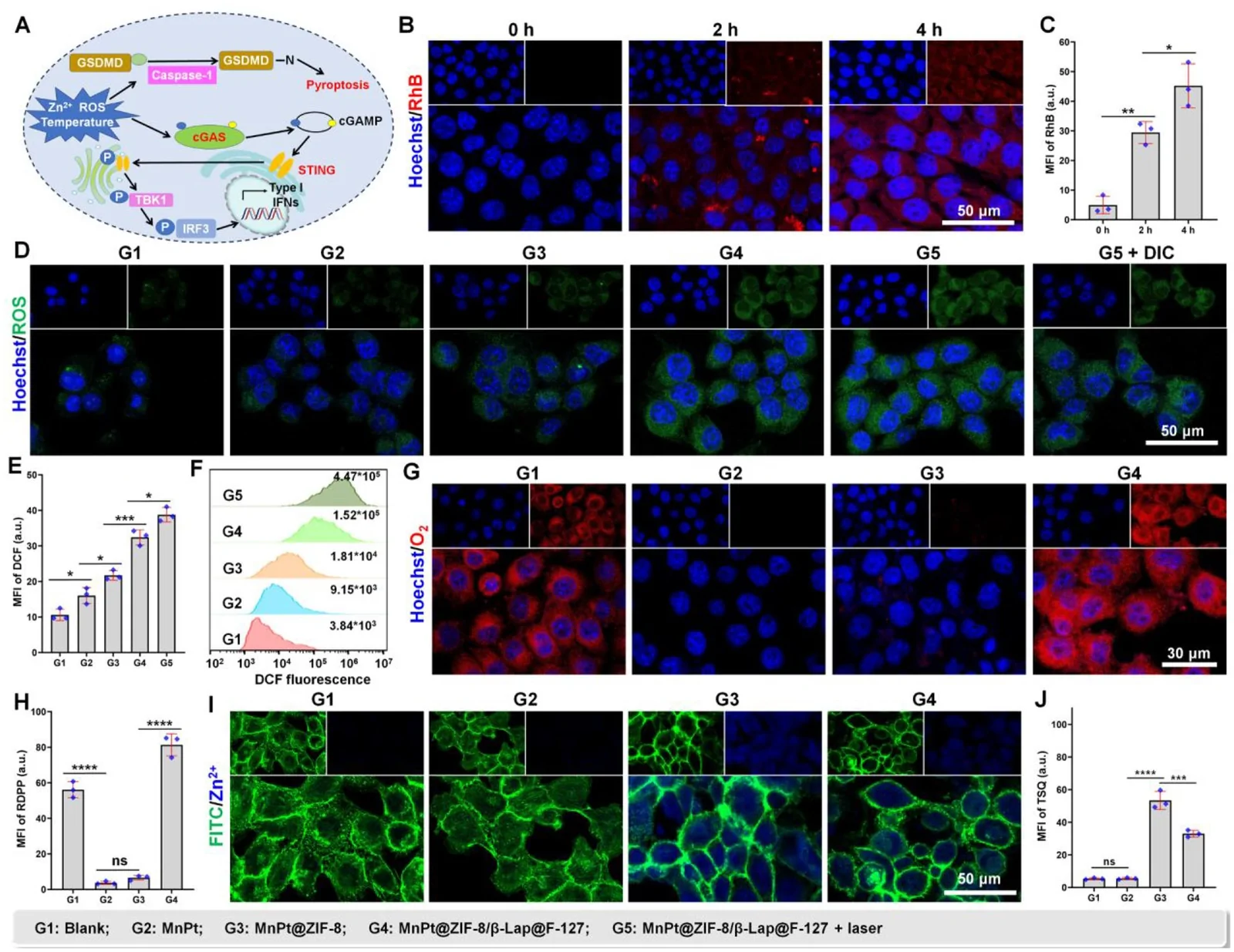
(**A**) Schematic illustration of Zn^2+^/ROS-activated cGAS-STING and pyroptosis pathways. (**B and C**) CLSM images and RhB fluorescence intensity analysis of RhB-labeled MnPt@ZIF-8/β-Lap@F-127 (*n* = 3). (**D and E**) CLSM images and DCF fluorescence intensity analysis of intracellular ROS (*n* = 3). (**F**) Flow cytometry analysis of DCF fluorescence. (**G and H**) CLSM images and RDPP fluorescence intensity analysis of intracellular O_2_ (*n* = 3). (**I and J**) CLSM images and TSQ fluorescence intensity analysis of intracellular Zn^2+^ (*n* = 3).

Prior to measuring the intracellular enzyme-like activity, the cellular uptake was first monitored by labeling labeling MnPt@ZIF-8/β-Lap@F-127 with RhB. The confocal laser scanning microscopy (CLSM) images revealed that the RhB fluorescence showed a progressive increase with extended co-incubation time (Figure 4B and C), indicating the efficient cellular uptake capability. It was well documented that NAD(P)H: quinone oxidoreductase 1 (NQO1) was highly expressed in 4T1 cells, and DIC acted as an inhibitor of NQO1 to confirm the enhanced ROS production of β-Lap [43, 44]. The intracellular ·OH level was verified using DCFH-DA, in which the produced ·OH can react with DCFH-DA to form 2′,7′-dichlorofluorescein (DCF) with green fluorescence. In comparison with the blank group, the DCF fluorescence gradually enhanced in the groups of MnPt, MnPt@ZIF-8, MnPt@ZIF-8/β-Lap@F-127 and MnPt@ZIF-8/β-Lap@F-127 + laser (Figure 4D and E), in which the Fenton-like reaction of Mn^2+^, the induced mitochondria damage of Zn^2+^, the H_2_O_2_ generation of β-Lap were responsible for the ·OH production [45, 46] and the temperature elevation further amplified the enzyme-like activity. By adding DIC, the green fluorescence was significantly reduced, indicating an effective activity inhibition of NQO1 (Figure 4D). Moreover, the laser irradiation further enhanced the intracellular ·OH level, owing to. In addition, the quantitative DCF fluorescence was analyzed using flow cytometry, in which the strongest DCF fluorescence was observed in the G5 group (Figure 4F), confirming the superior ·OH production capability of MnPt@ZIF-8/β-Lap@F-127 with laser irradiation.

The intracellular O_2_ concentration was monitored, in which the produced O_2_ can quench the RDPP fluorescence (Figure 4G and H). In the blank group, the strongest RDPP fluorescence was observed, and the RDPP fluorescence almost disappeared in the groups of MnPt and MnPt@ZIF-8, owing to the O_2_ production of MnPt by catalyzing H_2_O_2_. However, the RDPP fluorescence was restored in the MnPt@ZIF-8/β-Lap@F-127 group, which was attributed to the O_2_ consumption in the redox reaction between β-Lap and NQO1. The intracellular Zn^2+^ level was monitored using Zn^2+^-specific TSQ probe. The blue fluorescence was markedly enhanced in the MnPt@ZIF-8 and MnPt@ZIF-8/β-Lap@F-127 groups (Figure 4I and J), in which the ZIF-8 degradation resulted in the Zn^2+^ accumulation.

GSH can undergo redox reaction with ROS, and the excessive GSH will severely limit the catalytic activity. By increasing sample concentration, the intracellular GSH level gradually decreased, in which the proportion of GSH consumption exceeded 60% (Figure 5A), verifying the good intracellular GSH depletion ability of MnPt@ZIF-8/β-Lap@F-127. Using the MTT assay, the cytotoxicity of MnPt, MnPt@ZIF-8 and MnPt@ZIF-8/β-Lap@F-127 was assessed, in which the viability of MCF-10A cells remained above 90% (Figure 5B), thereby confirming their low cytotoxicity. The *in vitro* combination efficacy was also evaluated using MTT assay. As illustrated in Figure 5C, the viability of 4T1 cells treated with MnPt, MnPt@ZIF-8 and MnPt@ZIF-8/β-Lap@F-127 was 86.6%, 53.2% and 34.1%, and further decreased to 24.5% in the group of MnPt@ZIF-8/β-Lap@F-127 with laser irradiation. Moreover, the CAM/PI staining exhibited the similar results with MTT assay (Figure 5D), demonstrating the good *in vitro* efficacy of MnPt@ZIF-8/β-Lap@F-127 with laser irradiation.

**Figure 5:**
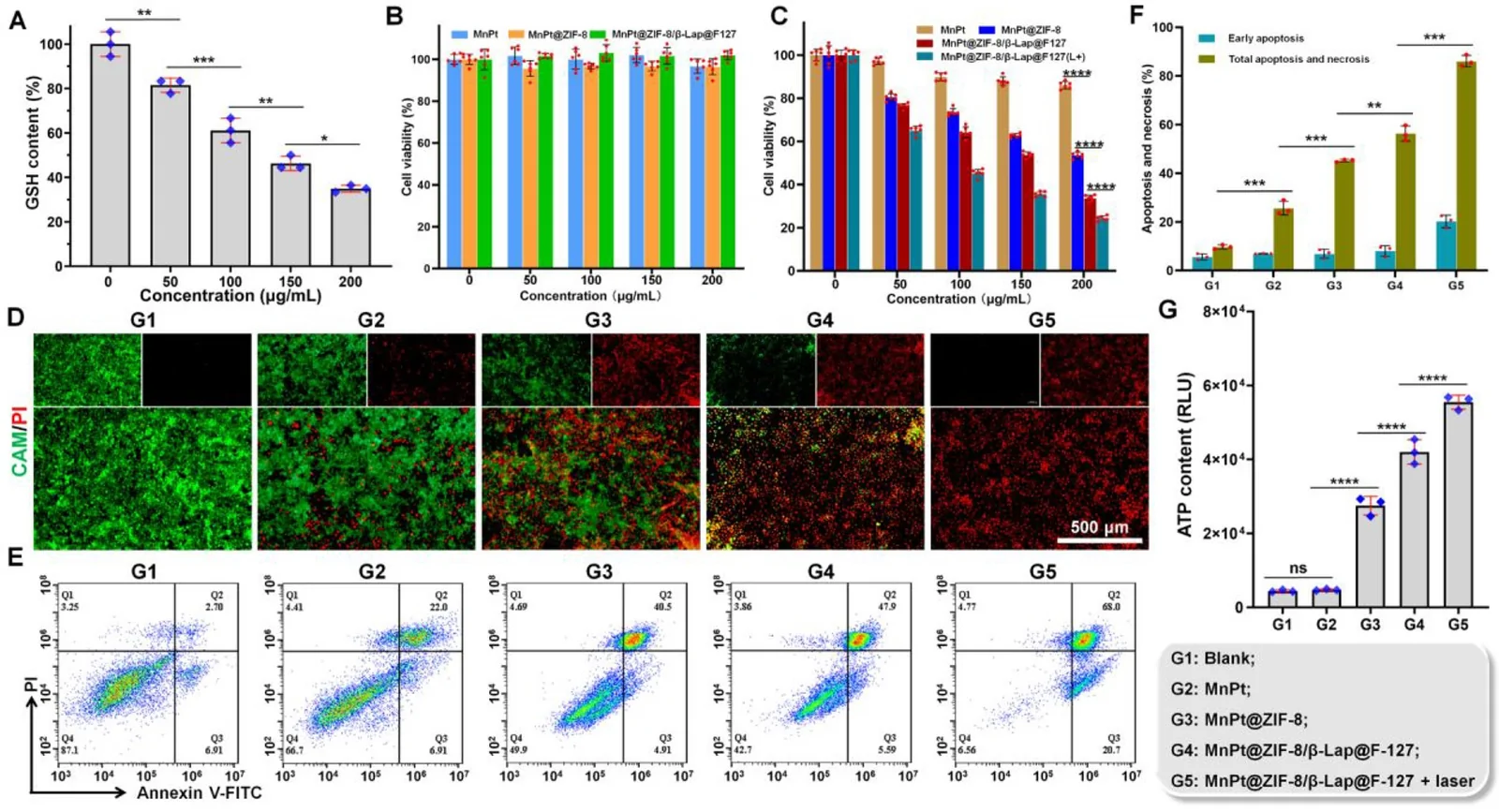
(**A**) Intracellular GSH content (*n* = 3). (**B**) MCF-10A cells survival rates with different samples (*n* = 6). (**C**) Survival rates of 4T1 cells with different treatments (*n* = 6). (**D**) CAM/PI staining images of 4T1 cells with different treatments. (**E and F**) Flow cytometry analysis and quantitative statistics of apoptosis (*n* = 3). (**G**) Extracellular ATP content (*n* = 3).

Using flow cytometry, the cell apoptosis and necrosis were analyzed (Figure 5E and F). From G1 to G4 groups, the early apoptosis rate was 5.6%, 6.9%, 6.9% and 8.1%, but the total apoptosis/necrosis rate increased from 9.6% to 25.7%, 45.2% and 56.3%. Especially in the G5 group, both of the early apoptosis and total apoptosis/necrosis rates greatly elevated to 20.2% and 86.1%, which was attributed to the enhanced catalytic reactions induced by laser irradiation. As a crucial signaling molecule, ATP played a vital role in recruiting dendritic cells (DCs) and promoting their maturation. Moreover, during ICD, ATP can be released to extracellular space, which aggravated the apoptosis and promoted the immune activation. Compared with G1 and G2 groups, the ATP level in G3 group significantly increased and further increased in G4 and G5 groups (Figure 5G), which verified the Zn^2+^-mediated ICD initiation and the ROS/photothermal-enhanced immune activation.

### Intracellular mitochondrial damage and immune activation

The mitochondrial damage can specifically be evidenced by a reduced MMP, which was visualized using a JC-1 probe (Figure 6A and B). In comparison to the blank group, the slight green fluorescence was observed in the MnPt and MnPt@ZIF-8 groups. However, in the groups of MnPt@ZIF-8/β-Lap@F-127 and MnPt@ZIF-8/β-Lap@F-127 with laser irradiation, the green fluorescence was greatly enhanced, confirming the effective accumulation of JC-1 monomer. Moreover, using flow cytometry, the statistical JC-1 monomer/aggregate was analyzed. From G1 to G5 groups, the percentage of JC-1 aggregate decreased significantly from 97.8% to 3.88%, exhibiting the considerable reduction of JC-1 aggregate (Figure 6C and D). This phenomenon represented the MMP reduction, which was collectively contributed by the ROS accumulation and Zn^2+^-mediated mitochondrial damage, demonstrating the mitochondrial damage ability of MnPt@ZIF-8/β-Lap@F-127.

**Figure 6:**
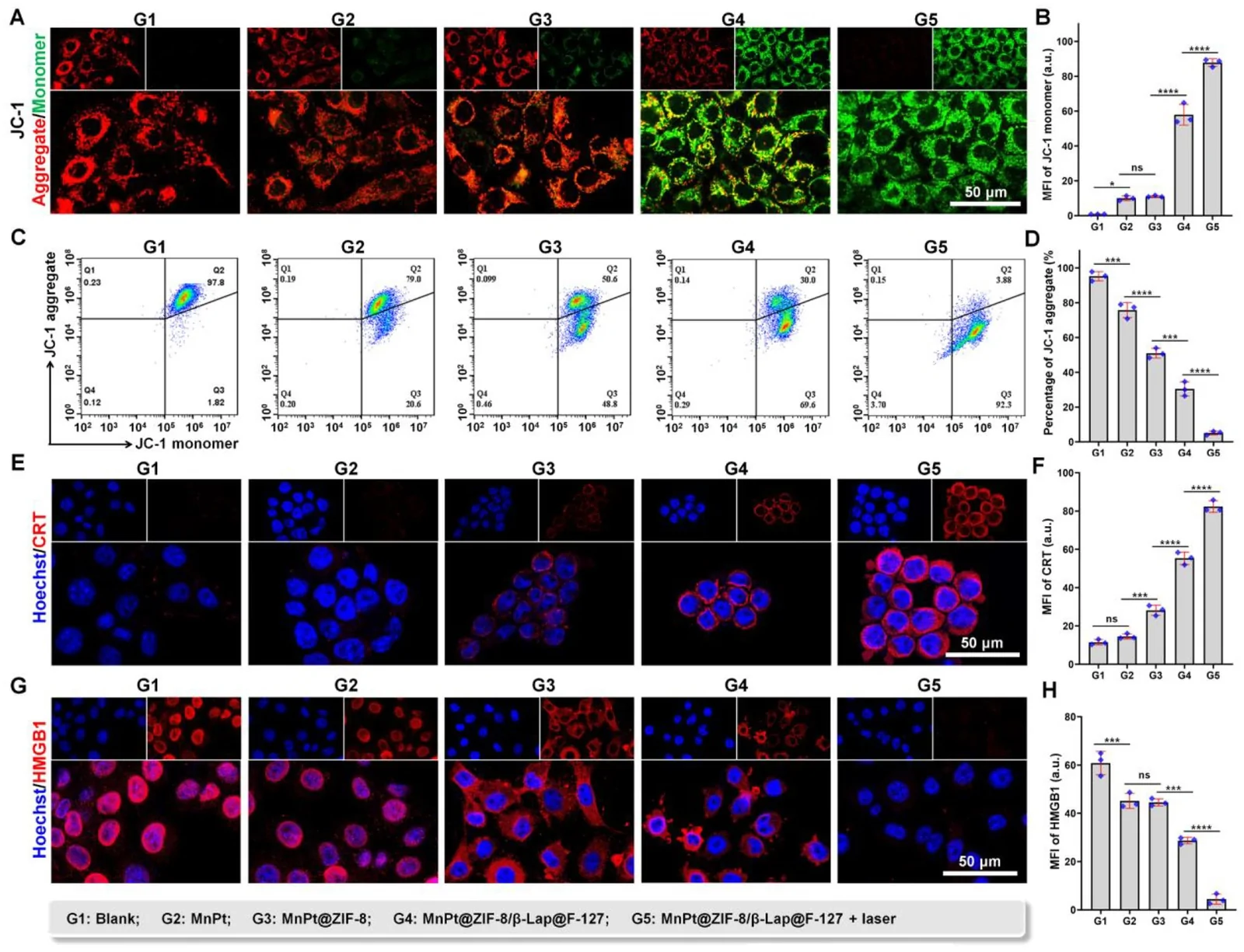
(**A and B**) CLSM images and fluorescence intensity analysis of JC-1 (*n* = 3). (**C and D**) Flow cytometry and percentage analysis of JC-1 aggregate (*n* = 3). (**E and F**) CLSM images and fluorescence intensity analysis of CRT (*n* = 3). (**G and H**) CLSM images and fluorescence intensity analysis of HMGB1 (*n* = 3).

During ICD, DAMPs (such as CRT exposure, HMGB1 release and ATP secretion) were activated, and an immune response was elicited. Using immune fluorescence technique, the DAMPs were measured. In the blank and MnPt groups, the CRT expression was relatively low and significantly enhanced in the groups of MnPt@ZIF-8, MnPt@ZIF-8/β-Lap@F-127 and MnPt@ZIF-8/β-Lap@F-127 with laser irradiation (Figure 6E and F). Conversely, from G1 to G5, the HMGB1 expression gradually decreased but underwent the translocation from the nucleus to the cytoplasm and ultimately to extracellular space (Figure 6G and H). The CRT exposure and HMGB1 release can be attributed to the combination of Zn^2+^/ROS/photothermal, confirming the ICD initiation and immune activation.

It was previously reported that Zn^2+^ can activate the cGAS-STING pathway and induce caspase-1-dependent pyroptosis [47, 48]. The activation of pyroptosis and cGAS-STING pathway induced by MnPt@ZIF-8/β-Lap@F-127 was demonstrated by measuring the production of tumor antigens. As a key protein of pyroptosis pathway, GSDMD is sheared to form GSDMD-N, which can rupture the cell membrane and cause the leakage of cell contents, thereby activating the immune response. Compared with G1 and G2 groups, the fluorescence intensity and expression level of GSDMD-N greatly increased in G3-G5 groups (Figure 7A and B), which verified the Zn^2+^/ROS/photothermal-mediated pyroptosis activation. For cGAS-STING pathway, Zn^2+^ is detected by cGAS to form cGAMP, thereby activating STING, followed by the TBK1 and IRF3 activation, thus further induced the gene expression of immune-related cytokines. Compared with G1 and G2 groups, the expression levels of p-STING, p-TBK1 and p-IRF3 proteins significantly increased in the G3 group and further increased in the G4 and G5 groups (Figure 7C–H), which verified the activation and enhancement of cGAS-STING pathway. The above results proved that the MnPt@ZIF-8/β-Lap@F-127 nanozyme activated the cGAS-STING pathway and induced pyroptosis thereby confirming its capacity to activate the immune response.

**Figure 7:**
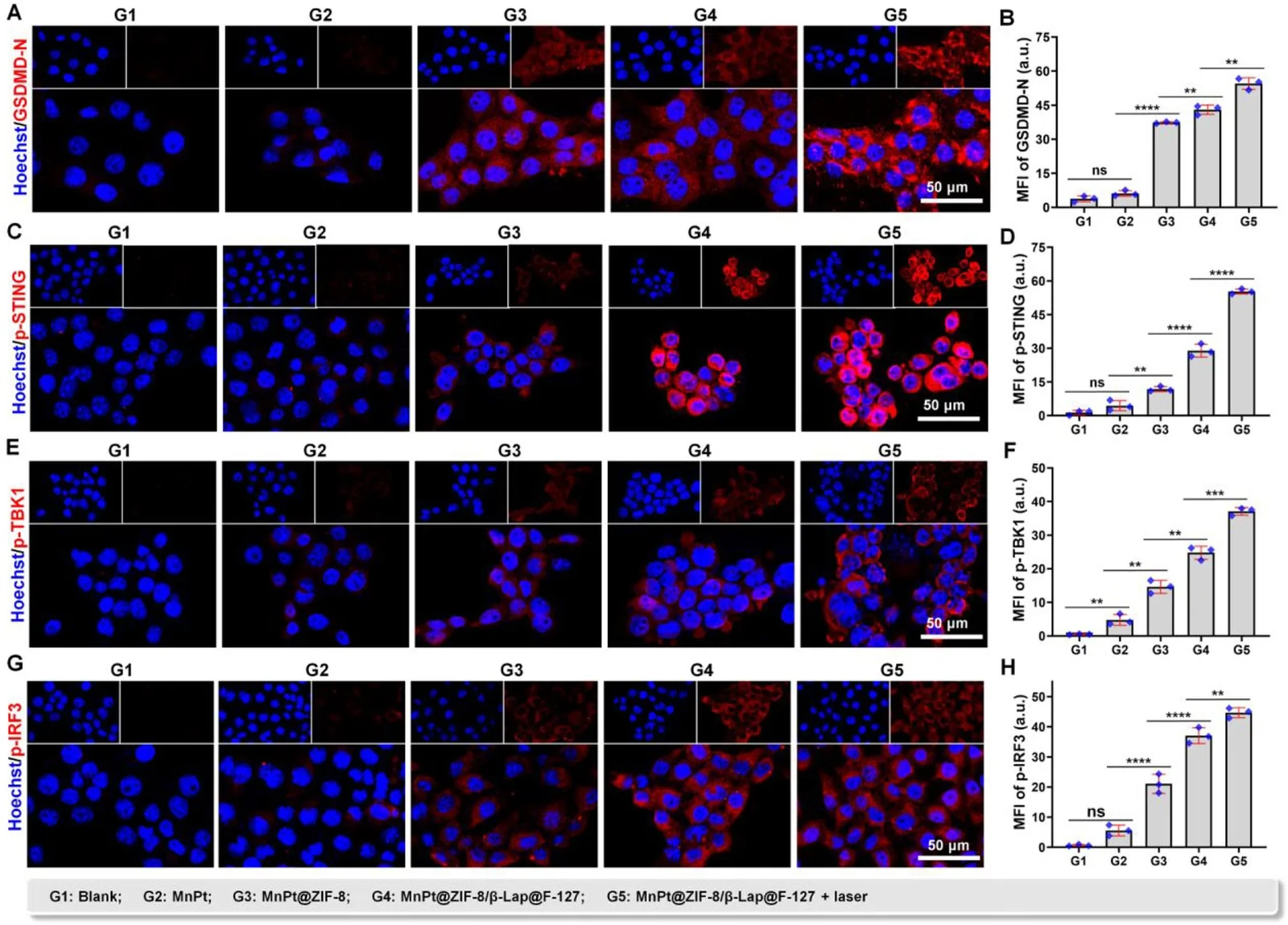
**(A and B)** CLSM images and fluorescence intensity analysis of GSDMD-N (*n* = 3). (**C and D**) CLSM images and fluorescence intensity analysis of p-STING (*n* = 3). (**E and F**) CLSM images and fluorescence intensity analysis of p-TBK1 (*n* = 3). (**G and H**) CLSM images and fluorescence intensity analysis of p-IRF3 (*n* = 3).

### *In vivo* efficacy and metastatic inhibition

First, the *in vivo* biocompatibility of MnPt@ZIF-8/β-Lap@F-127 was evaluated by measuring hemolysis rate, blood routine and biochemistry and observing H&E staining of organs. When the MnPt@ZIF-8/β-Lap@F-127 concentration reached 500 μg/mL, the hemolysis rate was still below 5%, confirming the blood biocompatibility (Supplementary Figure S9). Compared with the saline group, there was no significant change in blood biochemistry and blood routine indexes (Supplementary Figure S10), and no obvious pathological changes were found in the H&E staining images of heart, liver, spleen, lung and kidney (Supplementary Figure S11), confirming the biosafety of MnPt@ZIF-8/β-Lap@F-127. The protocols of animal experiments are shown in Figure 8A. Based on the NIR-light absorption capability of MnPt, the PA imaging capability of MnPt@ZIF-8/β-Lap@F-127 was assessed under an 808 nm laser irradiation, in which the PA signal got stronger by increasing the sample concentration (Figure 8B and Supplementary Figure S12). After intravenous injection, the PA signal of tumor site reached a maximum at 12 h (Figure 8C and Supplementary Figure S13). In addition, 12 h after injection, the temperature at the tumor site was recorded under an 808 nm laser irradiation (Figure 8D and E). It was observed that the temperature of the tumor site in the saline group rose to 38.6°C but reached 45.1°C in the MnPt@ZIF-8/β-Lap@F-127 group, confirming the mild photothermal effect. The PA imaging and photothermal measurements at the tumor site demonstrated the effective accumulation of MnPt@ZIF-8/β-Lap@F-127.

**Figure 8:**
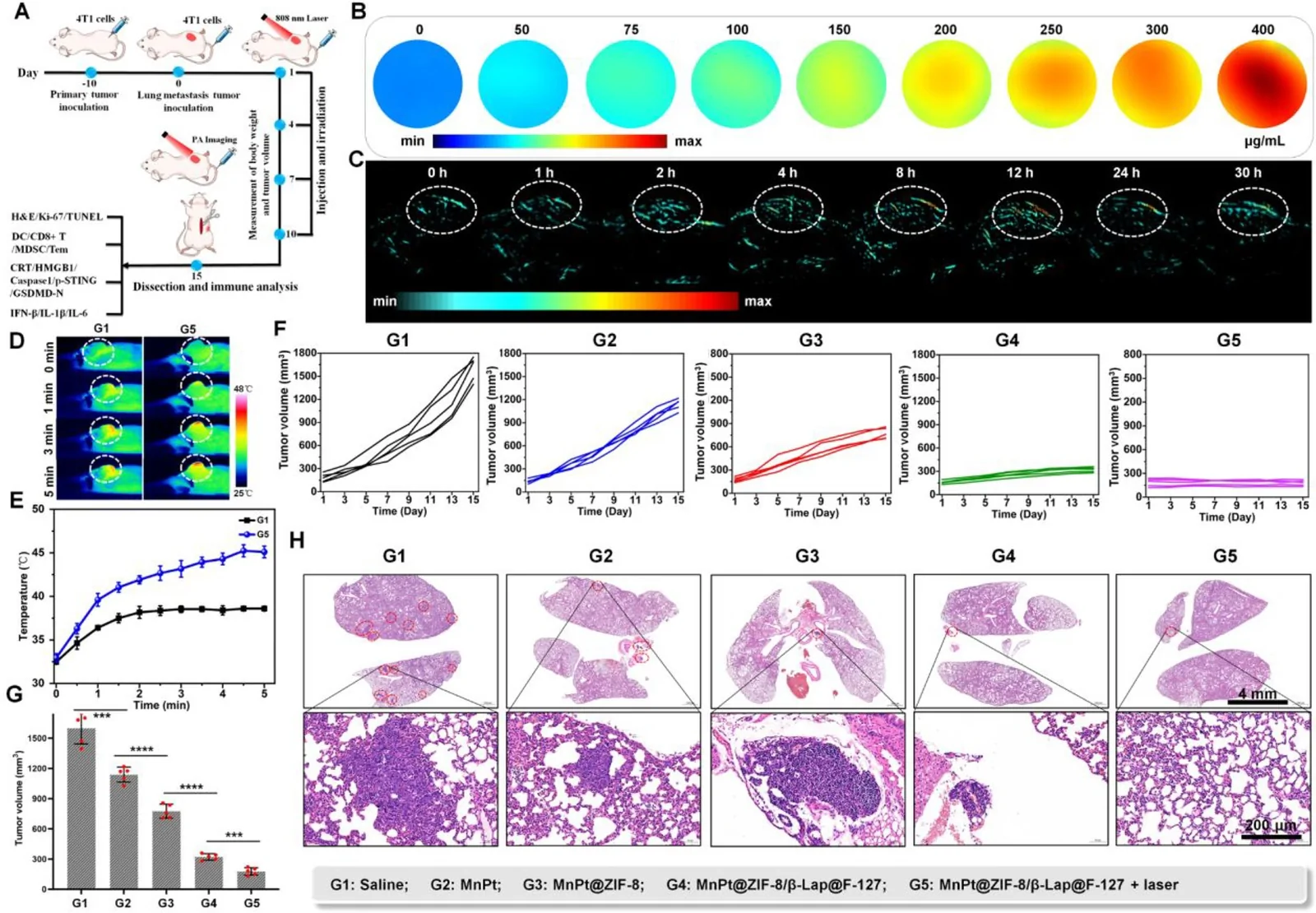
(**A**) Schematic illustration of animal experiment process. (**B**) PA signal images of MnPt@ZIF-8/β-Lap@F-127 with different concentrations. (**C**) PA imaging of MnPt@ZIF-8/β-Lap@F-127 at tumors with intravenous injection. (**D and E**) Infrared thermal imaging and temperature curves (*n* = 5). (**F**) Tumor volume curves. (**G**) Tumor volume histogram (*n* = 5). (**H**) H&E staining of lung.

Subsequently, the combination immunotherapy toward primary tumor and lung metastasis was evaluated throughout 15-days treatment. In the G1-G5 groups, the tumor volume increased to 1700 mm^3^, 1130 mm^3^, 770 mm^3^, 320 mm^3^ and 117 mm^3^, respectively (Figure 8F and G), in which the MnPt@ZIF-8/β-Lap@F-127 with laser irradiation exhibited a significant growth inhibition for primary tumor. Moreover, by observing the H&E staining images of lungs, many metastatic nodules were found in the blank group, and some metastatic nodules were found in the G2-G4 groups, but no metastatic nodule was found in the G5 group (Figure 8H), which indicated the MnPt@ZIF-8/β-Lap@F-127 with laser irradiation played an important role in inhibiting the tumor metastasis. All the results demonstrated the excellent combination immunotherapy efficacy of MnPt@ZIF-8/β-Lap@F-127 for primary and metastatic tumors.

To further verify the efficacy of MnPt@ZIF-8/β-Lap@F-127, the H&E/Ki-67/TUNEL staining was conducted (Figure 9A). Compared with the saline group, the tumor tissues in the MnPt group showed obvious DNA fragmentation, proliferation inhibition and apoptosis. With the introduction of Zn^2+^, β-Lap and laser irradiation, the apoptosis degree gradually deepened, indicating the effective antitumor efficacy of MnPt@ZIF-8/β-Lap@F-127. Moreover, in the group of MnPt@ZIF-8/β-Lap@F-127 with laser irradiation, the blood biochemistry and the body weight of mice was not significantly different from other groups (Figure 9B and C), no significant organ damage was observed (Figure 9D). All the data determined the validity and safety of combination immunotherapy of MnPt@ZIF-8/β-Lap@F-127.

**Figure 9:**
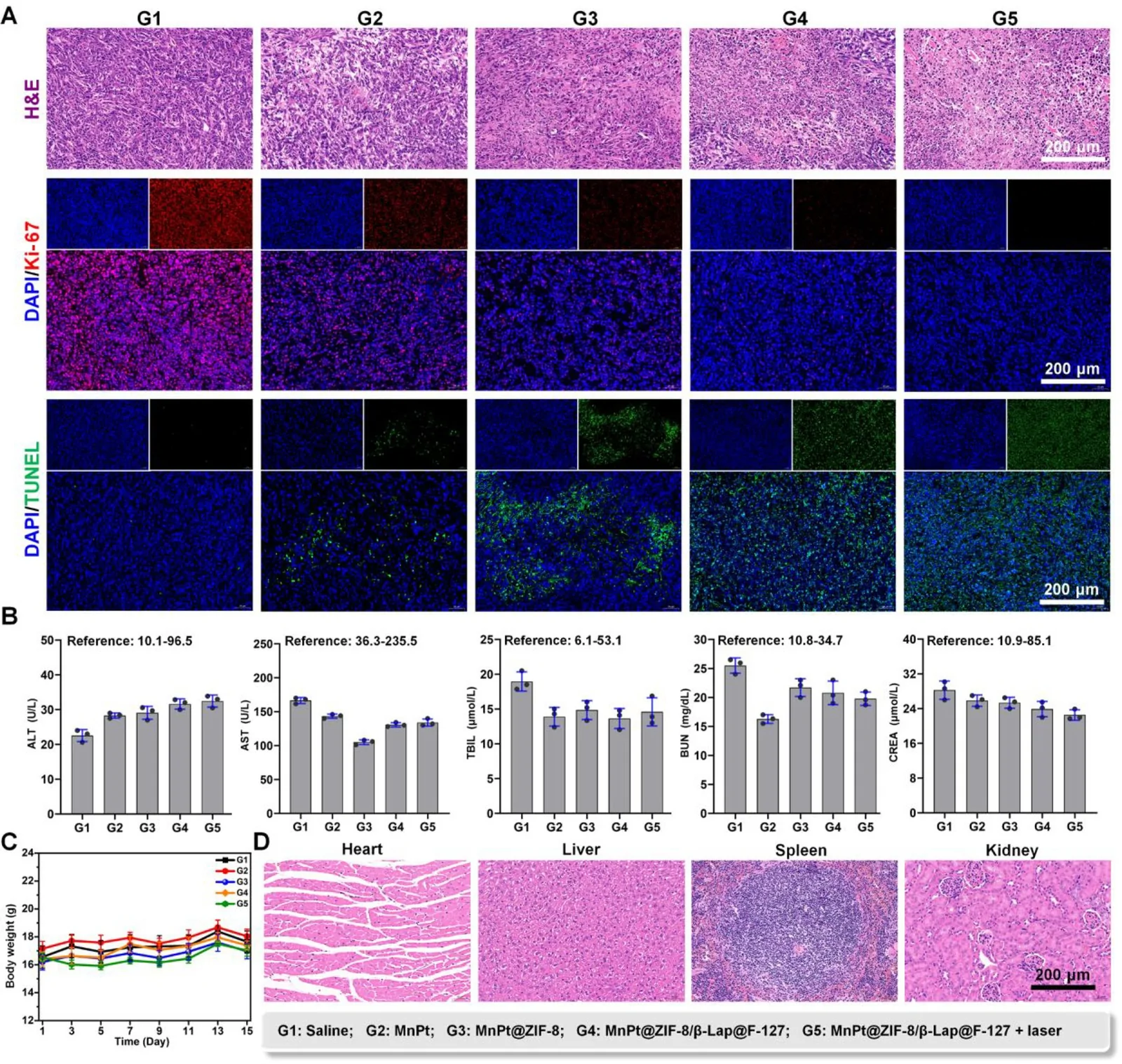
(**A**) H&E/Ki-67/TUNEL staining images of tumors. (**B**) Blood biochemical parameters (*n* = 3). (**C**) Body weight curves (*n* = 5). (**D**) H&E staining of organs in the G5 group.

### *In vivo* immune activation

To elucidate the efficacy and potential long-term immune mechanism of MnPt@ZIF-8/β-Lap@F-127, the immune response-related cells were detected using flow cytometry. As an important immune organ, the related immune cells in the spleen were first analyzed. The DC maturation played a crucial role in antitumor immunity, which was closely associated with antigen presentation. From G1 to G5 groups, the DC maturation was 48.1%, 53.9%, 57.7%, 66.8% and 71.8%, respectively (Figure 10A and B). Mature DCs facilitated the T cell activation through antigen presentation, and the proportion of CD8+ T cells in the G1-G5 groups significantly increased from 26.9%, 28.4%, 31.2%, 35.3% and 38.1% (Figure 10C and D). Effector memory T (Tem) cells and myeloid-derived suppressor cells (MDSCs) were two important signs in evaluating the long-term immune barrier and reversal of immunosuppressive TME. From G1 to G5 groups, the percentage of MDSCs was 76.5%, 64.5%, 57.8%, 46.3% and 38.9% (Figure 10E and F), and the percentage of Tem cells was 47.1%, 58.7%, 65.0%, 71.5% and 76.0% (Figure 10G and H), in which the highest percentage of Tem cells and the lowest percentage of MDSCs were observed in the G5 group, suggesting the establishment of a long-term immune barrier and the inhibition of MDSCs production by MnPt@ZIF-8/β-Lap@F-127 with laser irradiation.

**Figure 10:**
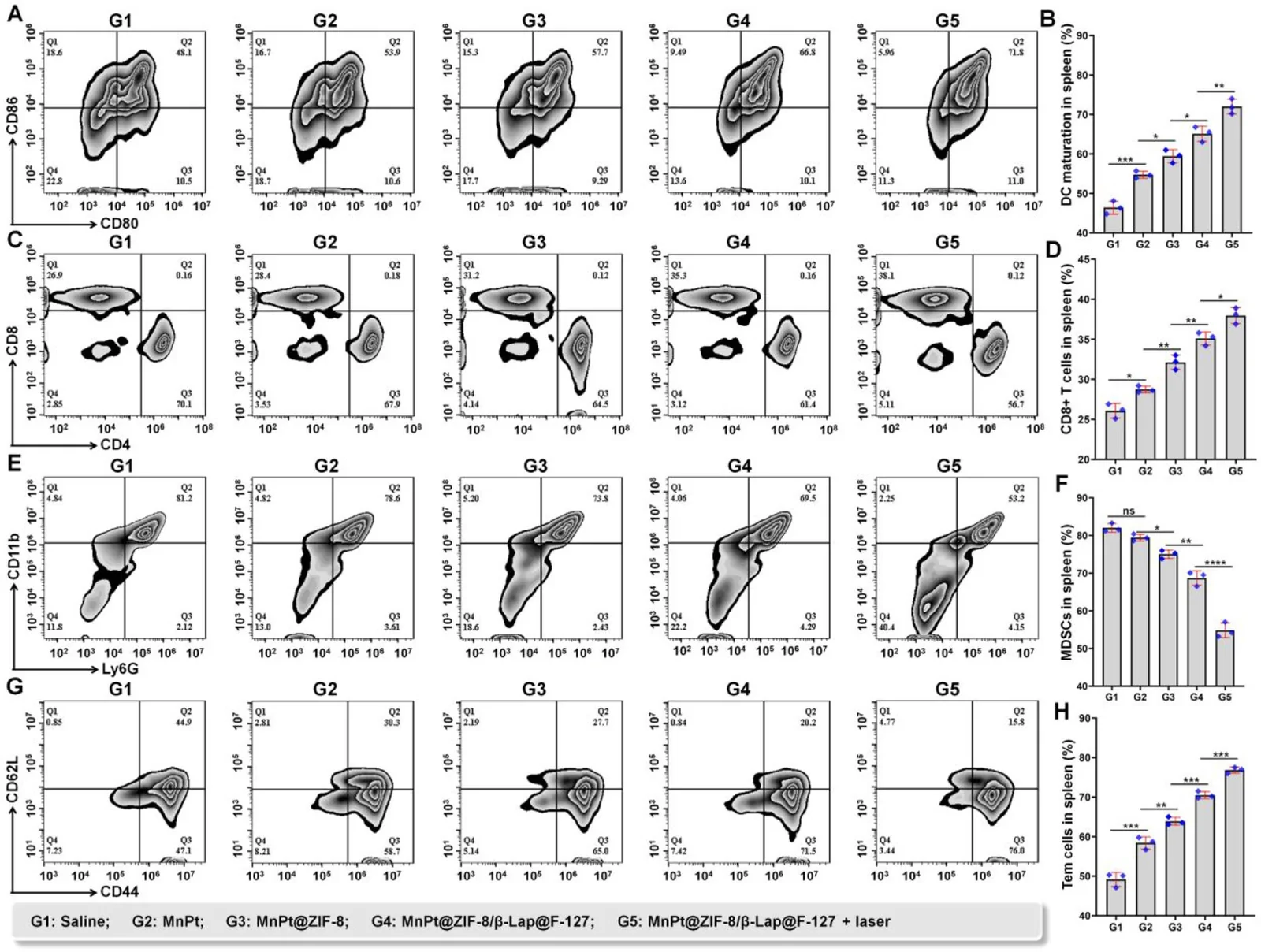
Flow cytometry analysis of immune activation in spleen (*n* = 3). (**A and B**) DC maturation. (**C and D**) CD8+ T cells. (**E and F**) MDSCs. (**G and H**) Tem cells.

Similar to the spleen, the immune-related cells in the tumor were also detected using flow cytometry. From G1 to G5 groups, the DC maturation was 49.8%, 52.7%, 78.2%, 80.5% and 82.4% (Figure 11A and B), the proportion of CD8+ T cells was 4.53%, 20.1%, 25.3%, 36.1% and 45.9% (Figure 11C and D), the percentage of MDSCs was 81.2%, 78.6%, 73.8%, 69.5% and 53.2% (Figure 11E and F), and the percentage of Tem cells was 69.2%, 75.7%, 78.5%, 81.2% and 84.5%, respectively (Figure 11G and H). All the results demonstrated that the MnPt@ZIF-8/β-Lap@F-127 with laser irradiation significantly increased the immune cell infiltration and inhibited the immunosuppressive cell production, thereby activating the cGAS-STING pathway and pyroptosis, also reversing the immunosuppressive TME.

**Figure 11:**
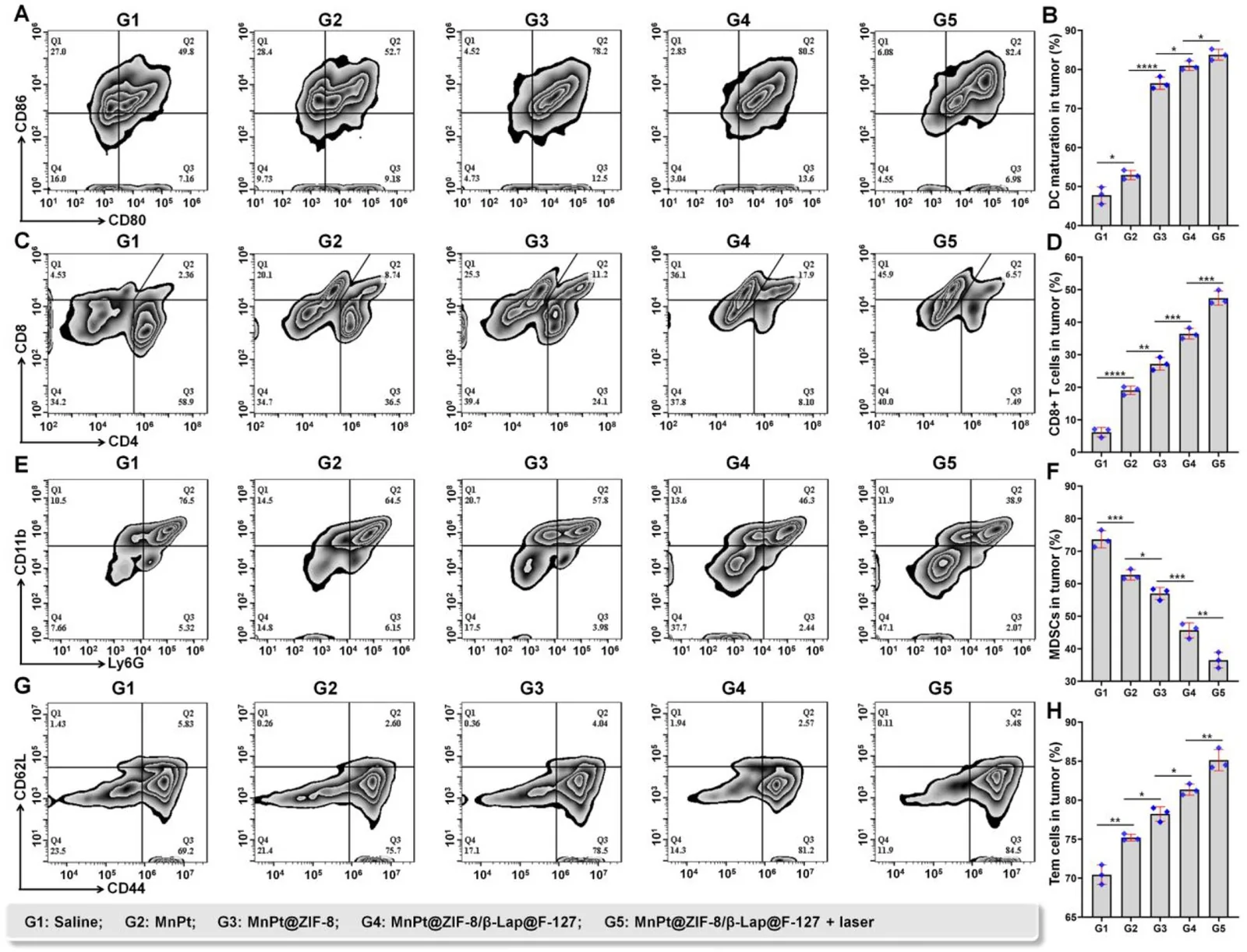
Flow cytometry analysis of immune activation in tumor (*n* = 3). (**A and B**) DC maturation. (**C and D**) CD8+ T cells. (**E and F**) MDSCs. (**G and H**) Tem cells.

To verify the activation mechanism of pyroptosis and cGAS-STING pathway, the expression levels of CRT, HMGB1, Caspase-1, GSDMD-N and p-STING in tumor tissues were assessed using immunofluorescence staining, and the release of IFN-β, IL-6 and IL-1β was also measured via ELISA. From G1 to G5 groups, the CRT exposure gradually enhanced (Figure 12A), and the HMGB1 was released across the nuclear and cytoplasmic membranes (Figure 12B). In comparison to the saline and MnPt groups, the Caspase-1 expression in the groups of MnPt@ZIF-8 and MnPt@ZIF-8/β-Lap@F-127 obviously decreased and significantly reduced in the group of MnPt@ZIF-8/β-Lap@F-127 with laser irradiation (Figure 12C), indicating its proteolytic activation. Moreover, the reduction of Caspase-1 expression subsequently cleaved the GSDMD and pro-IL-1β, which resulted in the generation of GSDMD-N and IL-1β (Figure 12D and F), thereby recruiting the immune cells and amplifying both local and systemic inflammatory responses.

**Figure 12:**
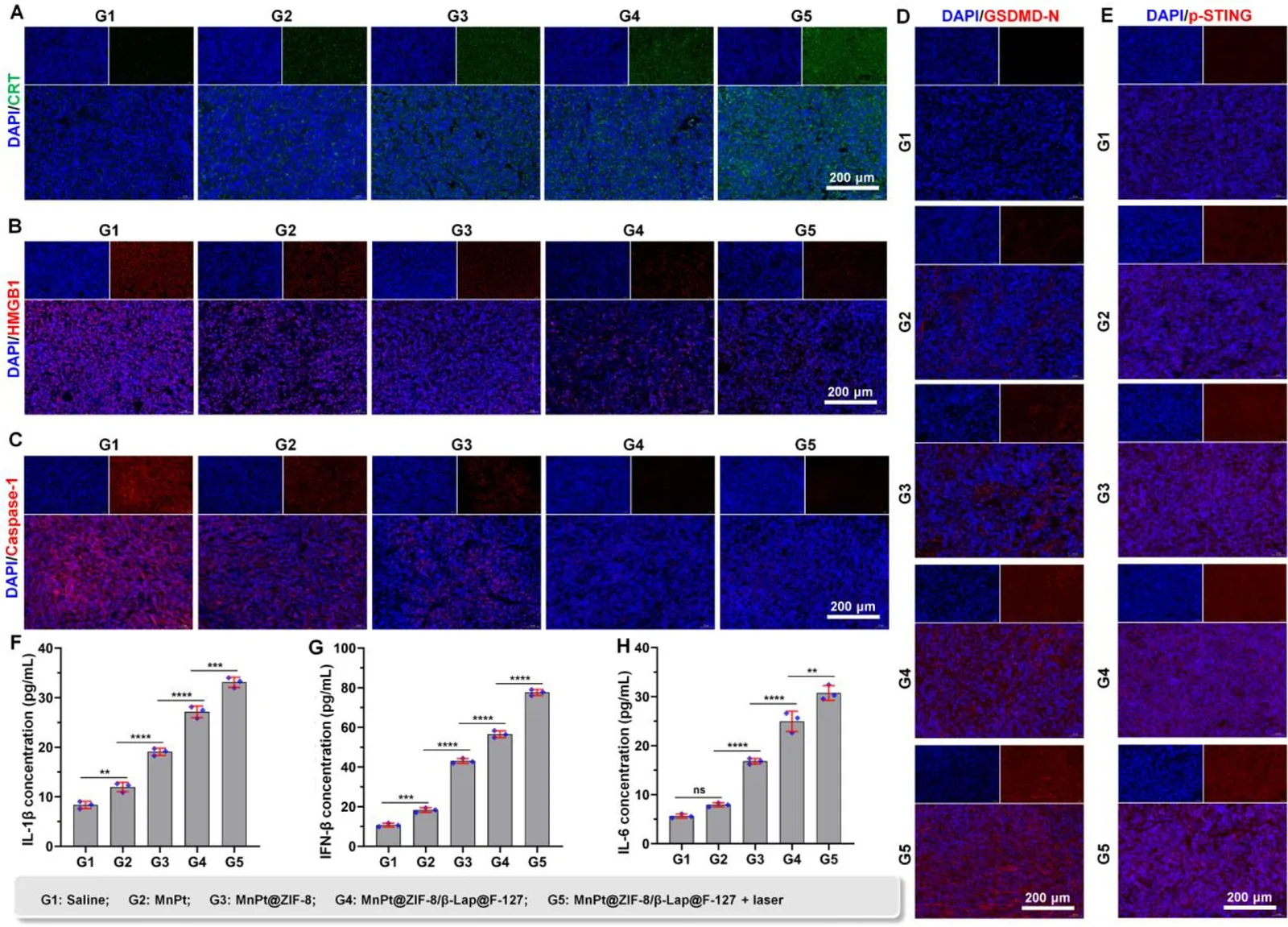
(**A**–**E**) Immunofluorescence images of CRT, HMGB1, caspase-1, GSDMD-N and p-STING in tumors. (**F**–**H**) ELISA assay of IL-1β, IFN-β and IL-6 levels.

The p-STING expression was directly related to the cGAS-STING pathway activation. As shown in Figure 12E, the strongest p-STING fluorescence was observed in the group of MnPt@ZIF-8/β-Lap@F-127 with laser irradiation, confirming the co-stimulation of ROS-induced mitochondrial stress and Zn^2+^-mediated cGAS-STING pathway. Furthermore, compared with the saline group, the levels of immune-stimulating cytokines in the group of MnPt@ZIF-8/β-Lap@F-127 with laser irradiation, such as IFN-β and IL-6, increased by nearly sevenfold and sixfold (Figure 12G and H), respectively. By following the results of immunofluorescence and ELISA, the MnPt@ZIF-8/β-Lap@F-127 nanozyme greatly activated the antitumor immune response through pyroptosis and cGAS-STING pathway.

## Conclusions

In summary, a multifunctional MnPt@ZIF-8/β-Lap@F-127 nanozyme was constructed for enhancing Zn^2+^-driven antitumor immunotherapy through photothermal/catalytic dual-strategy. The MnPt@ZIF-8/β-Lap@F-127 nanozyme exhibited multi-enzyme activities of POD/CAT/GPx-like, photothermal and PA imaging performances, achieving self-boosted catalysis and ROS amplification. In acidic TME, the ZIF-8 degradation accelerated the β-Lap and Zn^2+^ release. Moreover, the photothermal/catalytic combination activated Zn^2+^-triggered pyroptosis and cGAS-STING immune pathways, synergistically causing mitochondrial damage and amplifying immune response. The MnPt@ZIF-8/β-Lap@F-127 nanozyme increased the infiltration of cytotoxic T lymphocytes and Tem cells and inhibited the MDSCs production, establishing long-term antitumor immune memory and confirming remarkable efficacy for primary tumor and lung metastasis.

## Supplementary Material

rbag210_Supplementary_Data
