# Supplementary material for: MnPt@metal organic framework nanozyme with photothermal/catalytic dual-strategy enhances Zn2+-mediated immunotherapy through activating pyroptosis and cGAS-STING pathway
Source: Regen Biomater. 2026 Sep 21;13:rbag210. doi: 10.1093/rb/rbag210 (PMC13725651; doi:10.1093/rb/rbag210)
Supplement: Supplementary file 1 [file rbag210_supplementary_data.zip › Supporting Information.docx]

**Supporting Information**

**MnPt@metal organic framework nanozyme with photothermal/catalytic dual-strategy enhances Zn^2+^-mediated immunotherapy through activating pyroptosis and cGAS-STING pathway**

Zekai Li^a#^, Qi Zhao^b#^, Liye Wang^a^, Zhou Shen^b^, Feng Liu^a^, Jiamei Ma^b^, Lining Zhao^a*^, Xiaoping Yin^b*^, Leyong Zeng^a,b,c*^

*^a^College of Chemistry and Materials Science & College of Life Sciences, Chemical Biology Key Laboratory of Hebei Province,* *Hebei Research Center of the Basic Discipline of Synthetic Chemistry,* *Hebei University, Baoding, 071002, P.R. China.*

*^b^Hebei Key Laboratory of Precise Imaging of Inflammation Related Tumors & Department of Medical Oncology &* *Department of Radiology, Central Laboratory, Aﬃliated Hospital of Hebei University, Baoding 071000, P.R. China.*

*^c^Key Laboratory of Medicinal Chemistry and Molecular Diagnosis of the Ministry of Education, State Key Laboratory of New Pharmaceutical Preparations and Excipients, Hebei University, Baoding, 071002, P.R. China.*

*^#^These authors contributed equally to this work.*

**Corresponding authors. E-mail address*: zengly@hbu.cn (Leyong Zeng), hdfyrad@hbu.edu.cn (Xiaoping Yin), liningzhao@hbu.edu.cn (Lining Zhao)

**Figure S1** DLS curves of MnPt@ZIF-8/β-Lap@F-127 dispersed in different media. (A) H_2_O. (B) PBS (pH=7.4). (C) 1640 culture medium. (D) FBS.

**Figure S2** XRD patterns of MnPt and MnPt@ZIF-8/β-Lap@F-127.

**Figure S3** TEM images of MnPt@ZIF-8/β-Lap@F-127 in PBS with different pH values. (A) pH=7.4. (B) pH=6.5. (C) pH=5.5.

**Figure S4** Standard curve of β-Lap.

**Figure S5** Standard curve of Zn^2+^.

**Figure S6** Absorbance curves of TMB incubated with MnPt@ZIF-8/β-Lap@F-127 at different H_2_O_2_ concentrations.

**Figure S7** Temperature curves of MnPt@ZIF-8/β-Lap@F-127 with different laser power densities.

**Figure S8** Photothermal cycling curves of MnPt@ZIF-8/β-Lap@F-127.

**Figure S9** Hemolysis rate of MnPt@ZIF-8/β-Lap@F-127 at different concentrations (n=3).

**Figure S10** Blood routine and biochemistry indexes of healthy mice in different groups (n=3).

**Figure S11** H&E staining images of organs in different groups.

**Figure S12** PA signal intensity of MnPt@ZIF-8/β-Lap@F127 at different concentrations (n=3).

**Figure S13** PA signal intensity of tumor sites with MnPt@ZIF-8/β-Lap@F127 at different post-injection time (n=3).
