# Supplementary material for: MnPt@metal organic framework nanozyme with photothermal/catalytic dual-strategy enhances Zn2+-mediated immunotherapy through activating pyroptosis and cGAS-STING pathway
Source: Regen Biomater. 2026 Sep 21;13:rbag210. doi: 10.1093/rb/rbag210 (PMC13725651; doi:10.1093/rb/rbag210)
Supplement: Supplementary file 1 [file rbag210_supplementary_data.zip › Responses to Reviewers.docx]

Dear Prof. Ding and Reviewers,

We are respectfully submitting the revised manuscript (Manuscript ID RB-2026-679) to ***Regenerative Biomaterials*** entitled “*MnPt@metal organic framework nanozyme with photothermal/catalytic dual-strategy enhances Zn^2+^-mediated immunotherapy through activating pyroptosis and cGAS-STING pathway*”. We have revised carefully our manuscript according to the comments and advice of the reviewers. We would like to thank the reviewers for the helpful advice and comments, which increased our interests and will be helpful for our future work. The following is the response to the comments of Reviewers 1, 2, and 3 point by point.

**Reviewer: 1**

This manuscript constructs a core-shell MnPt@ZIF-8/β-Lap@F-127 multi-enzymatic nanozyme to boost Zn^2+^-mediated antitumor immunotherapy via combined catalytic and photothermal strategies. The nanoplatform integrates GPx/CAT/POD-like activities, photothermal and photoacoustic (PA) imaging capacity, and triggers mitochondrial damage, ICD, pyroptosis and cGAS-STING signaling activation, which remodels immunosuppressive tumor microenvironment, and achieves remarkable antitumor efficacy. The experimental design is rigorous, and the logical mechanism is clearly illustrated. The work exhibits important originality, and I recommend publication, but the following revisions are required before acceptance.

1. In Fig. 1h, the element symbols are unclear. Please amplify the font size.

**Response:** Thanks for your valuable advice and comment. In the revised manuscript, the font size of element symbols in **Figure 1**G and 1H were amplified.

**Figure 1** (G) TEM mapping images of MnPt. (H) TEM mapping images of MnPt@ZIF-8.

2. In this work, the authors calculate the loading/encapsulation/release of β-lap and the release of Zn^2+^ using a UV-Vis spectrometer. However, the standard curves of β-lap and Zn^2+^ are not shown. Please provide them.

**Response:** Thanks for your valuable comment. In the revised manuscript, the standard curves of β-Lap and Zn^2+^ are provided as **Figure S4** and **S5**.

**Figure S4** Standard curve of β-Lap.

**Figure S5** Standard curve of Zn^2+^.

3. As an important function of theranostic nanoplatform, the PA signals needed to be calculated. Please measure the values corresponded to Fig. 7b and c.

**Response:** Thanks for your valuable comment. In the revised manuscript, the PA signal intensity of MnPt@ZIF-8/β-Lap@F127 at different concentrations was calculated as **Figure S12**, and the PA signal intensity of tumor sites with MnPt@ZIF-8/β-Lap@F127 at different post-injection time was calculated as **Figure S13**.

**Figure S12** PA signal intensity of MnPt@ZIF-8/β-Lap@F127 at different concentrations (n=3).

**Figure S13** PA signal intensity of tumor sites with MnPt@ZIF-8/β-Lap@F127 at different post-injection time (n=3).

4. In Fig. 7g, the p-value annotation is misplaced. Please remark them.

**Response:** Thanks for your valuable advice and comment. In the revised manuscript, the p values were remarked in **Figure 7**G.

**Figure 7** (G) Tumor volume histogram (n=5).

5. In Fig. 8b, the authors measure the blood biochemistry data. Please add the reference range of normal values. Furthermore, In Fig. S6, please use a bar chart instead of a table, and also mark reference range.

**Response:** Thanks for your valuable advice and comment. In the revised manuscript, the reference range of normal values was marked in **Figure 8**B and **Figure S10**.

**Figure 8** (B) Blood biochemical parameters of 4T1 tumor mice in different groups (n=3).

**Figure S10** Blood routine and biochemistry indexes of healthy mice in different groups (n=3).

6. Some abbreviations should be defined, when they firstly appear, such as CRT, HMGB1. Please carefully check the full manuscript.

**Response:** Thanks for your valuable comment. We have carefully checked the manuscript, and the abbreviations were firstly defined, which was marked with blue font.

**Reviewer: 2**

This paper designs a MnPt@ZIF-8/β-Lap@F-127 composite nanozyme for combined pH-responsive Zn^2+^ and β-lapachone release, glutathione consumption, nanozyme catalysis, and 808 nm photothermal therapy. The authors propose that this system can amplify intracellular oxidative stress, disrupt mitochondrial function, induce tumor cells to undergo pyroptosis and immunogenic cell death, and activate the cGAS-STING signaling pathway, ultimately improving immune cell infiltration, inhibiting primary tumor growth and lung metastasis. This composite treatment strategy has certain value, but the system composition is relatively complex. The current control experiments are not sufficient to distinguish the individual roles of MnPt, ZIF-8-derived Zn^2+^, β-Lap, catalytic ROS and photothermal effects. The mechanisms of pyroptosis and cGAS-STING also lack direct evidence such as inhibitors or gene interventions. Therefore, I recommend major revisions.

1. The ability of β-Lap to generate ROS is highly dependent on NQO1. The authors should detect the expression and activity of NQO1 in 4T1 cells, and verify the ROS amplification mechanism mediated by β-Lap through NQO1 inhibitors or knockdown experiments.

**Response:** Thanks for your helpful advice and comment. It has been reported that NQO1 was high-expressed in 4T1 cells, and the related reference was cited as **Ref. 43** to support it. NQO1 was crucial for H_2_O_2_ generation of β-Lap, thereby enhancing ROS generation, while dicoumarol (DIC) acted as an inhibitor of NQO1 enzyme [44]. In the revised manuscript, an experimental group (G5 + DIC) was added to confirm the ROS amplification mechanism. As shown in **Figure 3D**, by adding DIC, the green fluorescence significantly reduced, indicating an effective activity inhibition of NQO1 enzyme. In the revised manuscript, the related description was added on **Page 9 and 20**, which was marked with red font.

43. Zhao X, Qiu L. Composite nanovesicles for enhanced chemodynamic cancer therapy via decitabine-mediated epigenetic reactivation. J Control Release 2026;394:114849.

44. Zhou L, Chen J, Sun Y, Chai K, Zhu Z, Wang C, Chen M, Han W, Hu X, Li R, Yao T, Li H, Dong C, Shi S. A self-amplified nanocatalytic system for achieving “1+1+1>3” chemodynamic therapy on triple negative breast cancer. J Nanobiotechnol 2021;19:1-13.

**Figure 3** (D) CLSM images of intracellular ROS (n=3).

2. It is suggested to set up a water bath control with the same temperature to determine whether the enhanced ROS generation and cell death caused by the laser are due to the overall temperature increase or the material-specific photothermal-catalytic coupling. Meanwhile, photothermal conversion efficiency, cycle stability, and temperature curves under different concentrations and powers should also be supplemented.

**Response:** Thanks for your valuable comment. The enhanced ROS generation was from photothermal-induced temperature elevation, which promoted enzyme-like activity. By setting different water bath temperature and different laser irradiation, the TMB absorbance incubated with MnPt@ZIF-8/β-Lap@F-127 was measured. As shown in **Figure R1** and **R2**, the TMB absorbance in the groups of water bath and laser irradiation significantly increased, confirming the temperature-dependent ROS generation. Moreover, the photothermal conversion efficiency, cycle stability, and temperature curves under different concentrations and power densities were shown in **Figure 2**K, 2L, S7, and S8.

**Figure R1** Absorbance curves of TMB incubated with MnPt@ZIF-8/β-Lap@F-127 at different water bath temperature.

**Figure R2** Absorbance curves of TMB incubated with MnPt@ZIF-8/β-Lap@F-127 at different laser power densities.

3. It is suggested to detect the β-Lap and metal ion release under pH 7.4, 6.5 conditions and in the presence of lysosomal pH in serum-containing medium. The structural changes of the MnPt core and ZIF-8 shell after degradation should be further characterized by TEM, DLS, elemental analysis and mass balance.

**Response:** Thanks for your valuable advice and comment. Owing to the lack of culture media with different pH values, the β-Lap release experiment in different culture media can not be carried out. By simulating different culture medium microenvironments, the β-Lap release in different PBS system also reflected similar results (pH=7.4: normal physiological environment; pH=6.5: tumor microenvironment; pH=5.5: lysosomal microenvironment). In the process of nanozyme degradation, the change in particle size lacked regularity. Therefore, only TEM images were provided as **Figure S2**. We hope you can accept our explanation.

**Figure S2**. TEM images of MnPt@ZIF-8/β-Lap@F-127 in PBS with different pH values. a) pH=7.4; b) pH=6.5; c) pH=5.5.

4. It should be clarified whether each "n" represents an independent biological replicate or a technical replicate. Specific statistical methods, multiple comparison corrections, and individual data points should be provided. Also, it should be explained whether the animal measurements and pathological analyses were conducted using a blinded method.

**Response:** Thanks for your valuable advice and comment. In this work, each "n" represents an independent biological replicate, and the animal measurements and pathological analyses were conducted using a blinded method. Moreover, statistical analysis was described in **Section 2.11**, and individual data points were also shown in statistical plots.

**Figure 4** (B) MCF-10A cells survival rates with different samples (n=6). (C) Survival rates of 4T1 cells with different treatments (n=6). (F) Flow cytometry quantitative statistics of apoptosis (n=3).

5. Some references regarding tumor therapy and cGAS-STING pathweay should be cited and discussed in the introduction section, including Nat. Commun. 2026, 17, 4756; Adv. Funct. Mater. 2026, 36, e19998; Biomaterials 329 (2026) 123987; Adv. Mater. 2021, 33, 2003458; Biomaterials 329 (2026) 123930; Biomaterials 325 (2026) 123559.

**Response:** Thanks for your valuable advice and comment. In the revised manuscript, the following papers were cited as **Ref. 4, 10, 21-23**.

4. Zhang Z, Yan L, Li W, Hu J, Wang Y, Dou H, Pan D, Shen L, Shi S, Geng B. D-π-A sensitized carbon dots as long-livedtype-I/II photosensitizers for NIR-excited hypoxia-regulated photodynamic therapy. Nat Commun 2026;17:4756.

10. Cai J, Shi S, Hu J, Zhang Z, Geng B, Pan D, Shen L. A stimuli-responsive cuproptosis switch boosts persistent immunotherapy for tumor eradication. Biomaterials 2026;329:123930.

21. Xiao L, Cai J, Wang Y, Hu J, Dou H, Pan D, Geng B, Cheng P, Shen L. Zr-based metal-organic framework dual-sensitized by carbon dot sonosensitizers and MnO_2-x_ nanozymes for cGAS-STING activation enhanced sonocatalytic-immunotherapy. Adv Funct Mater 2026;36:e19998.

22. Zhao J, Cai J, Hu J, Zhang Z, Liu Y, Pan D, Shen L, Geng B. Biodegradable hollow MnO_2_ decorated by carbon dots with cholesterol depletion capability for cascaded amplification of sono-immunotherapy. Biomaterials 2026;325:123559.

23. Hu J, Xiang Y, Zhang Z, Cai J, Wang Y, Dou H, Pan D, Shen L, Geng B. Atomic-valence engineering of a Mn(III)-tuned sonozyme system for multimodal tumor immunotherapy. Biomaterials 2026;329:123987.

**Reviewer: 3**

In this manuscript, authors developed a multifunctional MnPt@ZIF-8/β-Lap@F-127 nanozyme with multi-enzyme activity and photothermal and photoacoustic imaging properties. Within the TME, the MnPt@ZIF-8/β-Lap@F-127 nanozyme could produce a large amount of ROS to induced apoptosis and pyroptosis, and activated the cGAS- STING pathway and immunogenic cell death. Overall, this manuscript has a lot of hard work and achieved satisfactory results. Thus, I recommend its publication after major revision. There are some points to address:

1. Stability is important for biological applications. The authors should carry out the relevant experiments to investigate the stability of MnPt@ZIF-8/β-Lap@F-127.

**Response:** Thanks for your helpful advice and comment. To demonstrate the colloid stability, DLS curves of MnPt@ZIF-8/β-Lap@F-127 dispersed in different media (H_2_O, PBS (pH 7.4), 1640 culture medium and FBS) between 7 days were measured as **Figure S1**, and the related description was added on **Page 15**, which was marked with red font.

**Figure S1** DLS curves of MnPt@ZIF-8/β-Lap@F-127 dispersed in different media. (A) H_2_O. (B) PBS (pH=7.4). (C) 1640 culture medium. (D) FBS.

2. Mn^2+^, Zn^2+^, and photosensitizers in MnPt@ZIF-8/β-Lap@F-127 can all generate ROS within tumor cells and mediate mitochondrial oxidative stress. Which one plays the primary role?

**Response:** Thanks for your valuable comment. Firstly, I agreed with your viewpoint. Using the Fenton-like reaction of Mn^2+^ and Zn^2+^, and the POD-like activity of MnPt nanozyme, the ROS can be produced, and the mitochondrial oxidative stress can be triggered. However, the Fenton-like reaction of Mn^2+^ and Zn^2+^ required low pH value, which restricted the ROS generation. In this work, the ROS storm was mainly contributed by POD-like activity of MnPt, H_2_O_2_ generation of β-Lap, and photothermal effect, which collectively triggered mitochondrial oxidative stress.

3. Zn^2+^ can enhance the catalytic activity of cGAS, while Mn^2+^, derived from Mn^3+^, not only performs a similar function but also directly activates STING. Thus, the role of Mn^2+^ should also be characterized.

**Response:** Thanks for your valuable comment. Firstly, I agreed with your viewpoint. It is confirmed that Mn^2+^ plays an important role in activating cGAS-STING pathway, but the activation degree of cGAS-STING is positively correlated with Mn^2+^ concentration [Zhang Z, Yang J, Zhou Q, Zhong S, Luo J, Chai X, Liu J, Zhang X, Chang X, Wang H. The role and mechanism of the cGAS-STING pathway-mediated ROS in apoptosis and ferroptosis induced by manganese exposure. Redox Biol 2025;85:103761]. In the MnPt@ZIF-8/β-Lap@F-127 nanozyme, ZIF-8 can degrade to release Zn^2+^, but the MnPt alloy is stable (**Figure S2**), which restricted the Mn^2+^ release. Figure 6c-h also confirmed the weak cGAS-STING activation by single MnPt. We hope you can accept our explanation.

**Figure S2**. TEM images of MnPt@ZIF-8/β-Lap@F-127 in PBS with different pH values. a) pH=7.4; b) pH=6.5; c) pH=5.5.

4. In Fig. 6g, after IRF3 is phosphorylated, it should enter the nucleus rather than remain in the cytoplasm.

**Response:** Thanks for your valuable comment. Firstly, I agreed you’re your viewpoint. During activation of the cGAS-STING pathway, TBK1 mediates phosphorylation of IRF3 at the Golgi apparatus in the cytoplasm. Phosphorylated IRF3 subsequently undergoes homodimerization and translocates into the nucleus to drive type I interferon transcription. In our experiment, the main purpose is to characterize the IRF3 production in the cytoplasm, and the membrane breaker was not used. Therefore, the IRF3 fluorescence was only observed in the cytoplasm. In future work, we will investigate the nuclear IRF3 fluorescence by breaking nuclear membrane.

5. In Fig 7c, the tumor area should be circled for easier identification.

**Response:** Thanks for your carefully advice and comment. In the revised manuscript, the tumor areas were outlined with circular markers in **Figure 7**C and **7**D.

**Figure 7** (C) PA imaging of MnPt@ZIF-8/β-Lap@F-127 at tumors with intravenous injection.

**Figure 7** (D) Infrared thermal imaging of tumor areas.

6. The quantitative data of flow cytometry in Fig. 3f should be supplemented.

**Response:** Thanks for your carefully advice and comment. In the revised manuscript, the quantitative data of DCF fluorescence were marked in **Figure 3**F.

**Figure 3** (F) Flow cytometry analysis of DCF fluorescence.

7. The MnPt@ZIF-8/β-Lap@F-127 carries a positive charge and may damage the negatively charged cell membranes. Further measurements of hemolysis rate are necessary to investigate the blood compatibility.

**Response:** Thanks for your carefully advice and comment. In the revised manuscript, the hemolysis experiment was carried out. As shown in **Figure S9**, when the MnPt@ZIF-8/β-Lap@F-127 concentration reached 500 μg/mL, the hemolysis rate was still below 5%, confirming the good blood biocompatibility. In the revised manuscript, the experiment description and data explanation were added on **Page 12** and **27**, which was marked with red font.

**Figure S9**. Hemolysis rate of MnPt@ZIF-8/β-Lap@F-127 at different concentrations (n=3).

8. Please carefully check the spelling, grammar, and punctuation in the entire manuscript.

**Response:** Thanks for your carefully advice and comment. In the revised manuscript, the full manuscript was carefully checked and revised.

We hope that you could accept our revision and explanation, and we are looking forward to hearing from you.

Thanks for your comments and your careful work.

Best regards,

Yours sincerely,

Leyong Zeng
